# Factors Affecting Smallholders’ Perception of Climate Change in Eritrea

**DOI:** 10.1007/s00267-026-02383-7

**Published:** 2026-01-30

**Authors:** Tesfai Tsegai Kidane, Edward Lahiff, Trevor Donnellan, Seamus Crosse, Thia Hennessy

**Affiliations:** 1 Department of Food Business and Development, Cork University Business School, University College Cork, Cork, Ireland; 2 Teagasc, Rural Economy & Development Centre. Mellows Campus, Athenry, Co. Galway, Ireland; 3 School of Biological Earth and Environmental Sciences, Distillery Fields, North Mall, University College Cork, Cork, Ireland; 4 Cork University Business School, University College Cork, Cork, Ireland

**Keywords:** Climate change perception, Smallholder farmers, Extension services, Media exposure, Threat appraisal, Coping appraisal

## Abstract

Understanding how smallholder farmers perceive and respond to climate change is critical for informing adaptation strategies. Using survey data from 261 smallholder dairy farmers in Eritrea, this study applies the Model of Private Proactive Adaptation to Climate Change (MPPACC) to examine the perception of climate change. The objectives were to (i) develop and validate indices of perception, threat appraisal, and coping appraisal, (ii) explore factors associated with these indices, and (iii) examine associations among perception, threat appraisal, and coping appraisal. Reliability of constructs was assessed using Cronbach’s α, while validity was evaluated through principal axis factoring. A regression-based parallel mediation model with 5000 bootstrapped resamples was employed to estimate confidence intervals for the indirect effects. Results show that 93% of respondents linked climate change to shifting seasons, 76% to erratic rainfall, 88% to rising temperatures, and 41.6% identified greenhouse gas emissions as a cause. Perception scores were directly associated with extension services, education, and media exposure, and were negatively associated with higher altitude. The mediation analysis further showed indirect associations, with threat appraisal, though not coping appraisal, acting as an intervening variable in the relationships involving media exposure, heat stress and the interaction between age and farming experience. These findings highlight how institutional support, education, and communication efforts are associated with farmers’ climate change perception. By integrating socio-economic and environmental factors with cognitive processes within the MPPACC framework, this study offers insights relevant to strengthening smallholder resilience in Eritrea and comparable contexts.

## Introduction

Food and nutrition insecurity remains a pressing and persistent issue in Eastern Africa, where millions suffer acute shortages exacerbated by climate variability, conflict, economic instability, and fragile food systems (WFP, 2025; World Bank, 2025). Although incremental progress has been observed in recent decades, widespread hunger and malnutrition continue to threaten vulnerable populations. Structural institutional weaknesses, compounded by sustained food price inflation, further undermine household access to affordable and nutritious diets, challenging the resilience of agri-food systems (UN, 2024).

Smallholder farmers are central to achieving food and nutrition security and to promoting sustainable agri-food systems at local, regional, and global levels (Gomez y Paloma et al., 2020; Kapari et al., 2023). These farms contribute significantly to global food supply and price stability, yet they face considerable challenges, particularly in low-income countries. The main constraints are inadequate access to quality inputs, credit, extension services, weak market integration, poor rural infrastructure, and vulnerability to climate shocks (Touch et al., 2024).

Understanding farmers’ perceptions of climate change is critical, as these perceptions associate with how risks and opportunities are recognized, influence the willingness to adopt new practices, and reinforce effective adaptation and resilience in agri-food systems (Jellason et al., 2019; Ricart et al., 2022). For smallholder farmers in vulnerable agricultural systems, perception is particularly important because it drives behavioral responses to climatic shocks and informs long-term adaptation and risk management strategies (Molthan-Hill et al., 2025; Oriangi et al., 2024; Ricart et al., 2022).

Research has shown that farmers’ perceptions of climate change are associated not only with socio-economic and environmental factors (Bitew & Minale, 2025; Molthan-Hill et al., 2025; Oriangi et al., 2024; Zeleke et al., 2023) but also with underlying psychological processes that interact with these conditions (Doran et al., 2020; Shi et al., 2019; van Valkengoed et al., 2024). Grothmann & Patt’s (2005) Model of Private Proactive Adaptation to Climate Change (MPPACC) is a foundational contribution in this regard. Adapting the Protection Motivation Theory (PMT) to climate adaptation research, the model highlights threat appraisal and coping appraisal as important cognitive mechanisms associated with individuals’ adaptive responses to climate risks (Grothmann & Patt, 2005; Maddux & Rogers, 1983).

In this framework, threat appraisal refers to individuals’ evaluations of the severity of climate risks and their own vulnerability to those risks; while coping appraisal reflects beliefs about the effectiveness of adaptive measures and one’s capacity to implement them. These constructs have been applied in diverse empirical climate adaptation studies. For example, in African contexts, Kolapo et al., (2025) investigated psycho-cognitive influences among Nigerian smallholders, Maziya et al., (2024) examined psychological capital in South Africa, and Zobeidi et al. (2022) applied MPPACC to water scarcity responses in Ethiopia.

Eritrea, a country situated in the semi-arid region of the Horn of Africa, faces pronounced impacts from climate variability due to its challenging agro-ecological conditions and its heavy dependence on rainfed agriculture (Debesai et al., 2019). Despite this high vulnerability, the country remains significantly underrepresented in research on climate-risk perception and adaptation. Existing studies demonstrate that the factors influencing climate change perception differ widely across national and cultural contexts (Afsar et al., 2021; Troncarelli et al., 2023), suggesting that insights from other countries cannot simply be generalized to Eritrea. This lack of context-specific evidence limits the ability to design effective, locally grounded adaptation policies and constrains broader scientific understanding of how farmers in diverse environments appraise climate risks. Against this backdrop, the present study examines the extent to which environmental, institutional, and socioeconomic conditions are associated with farmers’ perceptions of climate change in Eritrea. In doing so, it also incorporates threat and coping appraisal mechanisms to explore how these cognitive processes relate to the broader set of contextual factors. The study therefore seeks to:

1. Develop and validate indices capturing smallholder farmers’ threat appraisal, coping appraisal, and climate-change perception.
2. Analyze the association between socioeconomic, institutional, and environmental factors and farmers’ climate-change perceptions, while also assessing how appraisal mechanisms relate to these factors.

## Study Area and Context

**Fig. 1:**
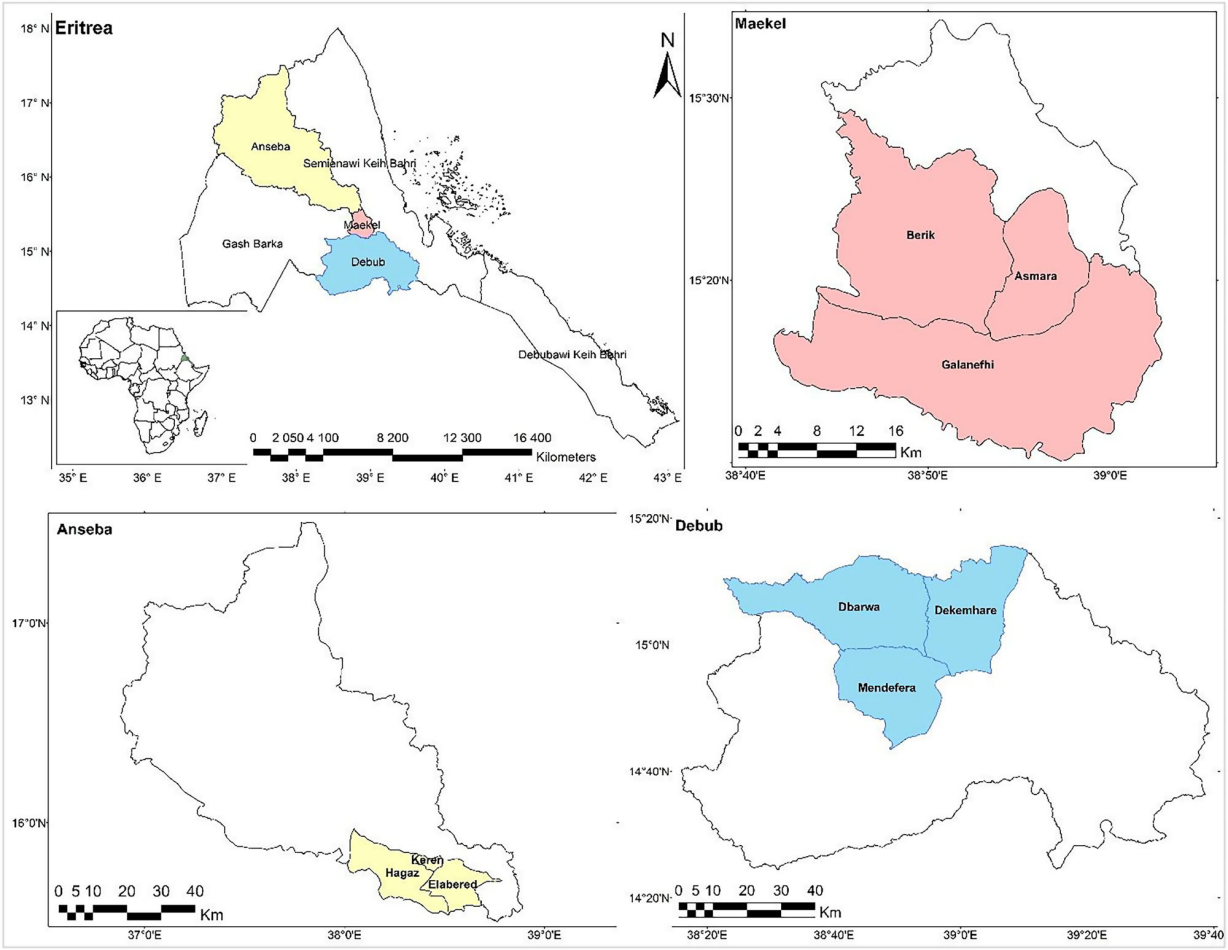
Study area

This study was conducted in three administrative zones in Eritrea, Maekel, Debub, and Anseba (Fig. 1), representing the highland and midland agroecological zones (Ministry of Land Water and Environment, 2012). Farming in these zones is predominantly carried out by smallholder mixed crop–livestock producers, with rain-fed agriculture as the primary livelihood source (MoA, 2024). These three zones account for about 60% of the dairy cattle population (Semereab Habtetsion, 2023) and more than 85% of the marketed milk in the country (Tesfay, 2024). This study was focused on smallholder dairy farmers managing herds of twenty or fewer animals, which make up 96% of the dairy farming in the study area (Tesfay, 2024).

Despite their ecological differences, the zones share major challenges, including high rainfall variability, land degradation, and limited institutional capacity for climate adaptation (Measho et al., 2019; T. Tesfay et al., 2018). Maekel, which includes the capital, Asmara, is characterized by intensive farming, relatively better market access, and scarcity of farm land (Haile et al., 2025). Debub features diverse agroecology and comparatively fertile soils, but remains highly dependent on variable rainfall (Kidane et al., 2019). Anseba is more arid and drought-prone, with chronic water scarcity and low-input production systems (Gebreyesus et al., 2022).

## Theoretical and Conceptual Framework

Troncarelli et al. (2023) note that most studies define climate change perception as awareness and interpretation of environmental changes and their impacts. Moreover, recent studies point to the relevance of socio-cognitive factors, including knowledge, beliefs, and subjective judgments, in understanding these perceptions (Kolapo et al., 2025; Okutan, 2025). In line with this, climate change perception, in this study, is defined as individuals’ interpretation of environmental changes, which reflects their direct experiences and the cognitive frameworks through which these changes are interpreted. This approach captures both the tangible manifestations of climate variability and the psychological dimensions associated with farmers’ interpretation of climate information. This is particularly relevant in countries of the Horn of Africa, where cultural norms, information accessibility, and traditional knowledge are associated with how climate change is understood (Debela et al., 2015).

Drawing on the attributes of climate change perception proposed by Hilbig (2024) and van Valkengoed et al. (2021), the study develops a Climate Change Perception Index capturing three core dimensions: the reality, causes, and consequences of climate change. The index includes four Likert-scale indicators (Table 1) that represent both observable environmental signals and farmers’ understanding of their drivers, drawing on prior research (Bitew & Minale, 2025; Ergun et al., 2024; Spence et al., 2011).

**Table 1:** Indicators used for index constructs of climate change perception

Indicators and Survey Statement	Justification	Category
Gradual Temperature Increase: Climate change means a gradual increase in temperature	Farmers recognize rising temperatures as a major indicator of climate change (Alhassan et al., 2025; Ricart et al., 2025)	Reality and consequence
Changes in Rainfall Patterns: Climate change means changes in rainfall patterns, such as droughts or floods	Most farmers perceive climate change as unpredictable rainfall and extreme events (Bitew & Minale, 2025; Dawid & Boka, 2025)	Reality and consequence
Changes in Seasons (Earlier/Delayed Planting): Climate change means changes in seasons, such as earlier or delayed planting	Farmers recognize seasonal shifts in rain-fed farming calendars as signals of climate change (Gemeda et al., 2023; Oriangi et al., 2024)	Reality and consequence
Rise in Greenhouse Gases (CO₂): The rise in greenhouse gases in the atmosphere is a major cause of climate change	Understanding the causes of climate change is an indicator of climate change perception(van Valkengoed et al., 2021) (Hilbig, 2024; van Valkengoed et al., 2021)	Cause

To examine the cognitive processes associated with these perceptions, the study draws on the Model of Private Proactive Adaptation to Climate Change (MPPACC) (Grothmann & Patt, 2005). Grounded in Protection Motivation Theory (Maddux & Rogers, 1983), MPPACC highlights two interrelated cognitive mechanisms, threat appraisal and coping appraisal, that influence how individuals interpret and respond to climate risks. The MPPACC framework has been applied in previous studies to explain socio-cognitive factors that explain climate adaptation behavior (Mitter et al., 2019; Y. Yang et al., 2025; Zobeidi et al., 2022). The model distinguishes between two cognitive appraisals: threat appraisal and coping appraisal (Grothmann & Patt, 2005). Building on the MPPACC framework, this study investigates the patterns of association between socioeconomic, institutional, and environmental factors and farmers’ threat and coping appraisals and perceptions. Thus, the analysis in this study emphasizes empirical interrelationships among these factors without assuming any sequential or causal ordering.

Following recent studies (Okutan, 2025; Sougué et al., 2024; Sungju Han, 2023), this research operationalizes threat and coping appraisal using indices derived from variables capturing farmers’ perceived climate risks and self-assessed adaptive capacity. The threat appraisal index includes six indicators (Table 2) reflecting farmers’ perceived risks from climate-related hazards (Bagagnan et al., 2019; Eitzinger et al., 2018). Previous research demonstrates a strong link between self-efficacy and adaptive behavior. For instance, Doran et al. (2020) found that prior land conservation practices were significantly associated with individuals’ self-assessed adaptive ability, while van Valkengoed & Steg (2019) identified perceived self-efficacy and outcome efficacy as the strongest predictors of adaptive action. Similarly, Zobeidi et al. (2022) reported self-efficacy as a significant determinant of adaptation behaviors, and Yang et al. (2024) reported that self-efficacy is a significant positive predictor of adaptive actions. Building on this empirical evidence, the present study uses farmers’ reported adaptive practices (Table 1) as a behavioral proxy for coping appraisal, reflecting both self-efficacy (confidence in one’s capacity to manage climate risks) and response efficacy (belief in the effectiveness of adaptive responses). This operationalization is consistent with PMT, which posits that adaptive behavior represents a tangible manifestation of individuals’ efficacy appraisals (Grothmann & Patt, 2005; Maddux & Rogers, 1983).

**Table 2:** Indicators used for index constructs of coping appraisal and threat appraisal

Appraisal	Indicators	Justification
Coping Appraisal	Use of adaptive strategies: manuring, crop rotation, fallowing, early maturing varieties, contour ploughing, on-farm terracing, drought-tolerant or high-yielding varieties, soil/water conservation measures, irrigation, seed storage, use of early warnings.	A farmer’s ability to implement adaptive strategies reflects self-efficacy and response efficacy (Doran et al., 2020; van Valkengoed & Steg, 2019)
Threat Appraisal	Perceived risk of crop losses due to short rains and rains during the harvest period. Insufficient rainfall, late onset of rains, uneven rainfall spatial and temporal distribution	Farmers’ perceptions of climatic risk reflect their exposure and evaluation of production losses (Atube et al., 2022; Eitzinger et al., 2018)

Within this broader socio-cognitive framework, socioeconomic and human-capital attributes are also associated with how individuals interpret climate risks. Variables such as age, gender, farming experience, education, and access to climate-related information are associated with both farmers’ cognitive appraisals and perceptions of climate change. Institutional factors, including access to agricultural extension services, membership in associations, proximity to markets, credit availability, and ownership of productive assets, further relate to how farmers understand and evaluate climate-related risks (Bitew & Minale, 2025; Dawid & Boka, 2025; Zeleke et al., 2023).

**Fig. 2:**
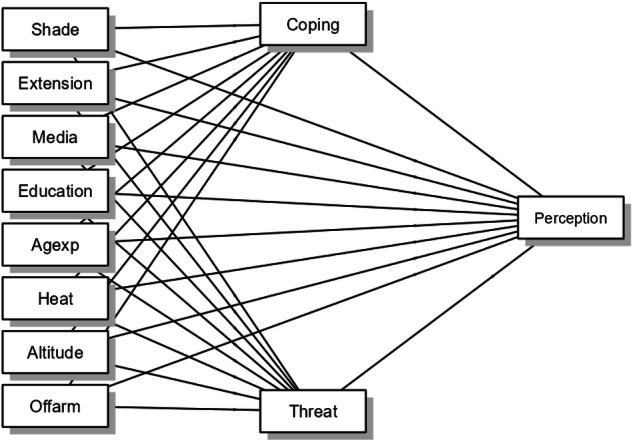
Conceptual framework of the study

As presented in the conceptual framework (Fig. 2), this study uses regression-based mediation models to examine how socio-economic, institutional, and environmental factors including ownership of improved dairy shade (Shade), access to extension services (Extension), media exposure (Media), educational attainment (Education), observation of heat stress (Heat), farm altitude (Altitude), combined age and farming experience (Agexp), and off-farm income sources (Offarm), relate to the cognitive appraisal constructs of coping appraisal (Coping) and threat appraisal (Threat), alongside climate change perception (Perception) as the outcome variable. While grounded in behavioral appraisal theory, the conceptual framework is intended as an analytical tool for examining these relationships and does not imply causal pathways.

## Data and Methods

This section describes the methodology used for data collection and analysis. It explains the sampling strategy and data collection procedures, followed by the validity and reliability checks applied in constructing the indices. Finally, it outlines the regression models employed to examine the direct and indirect associations with climate change perception.

### Primary Data

**Fig. 3:**
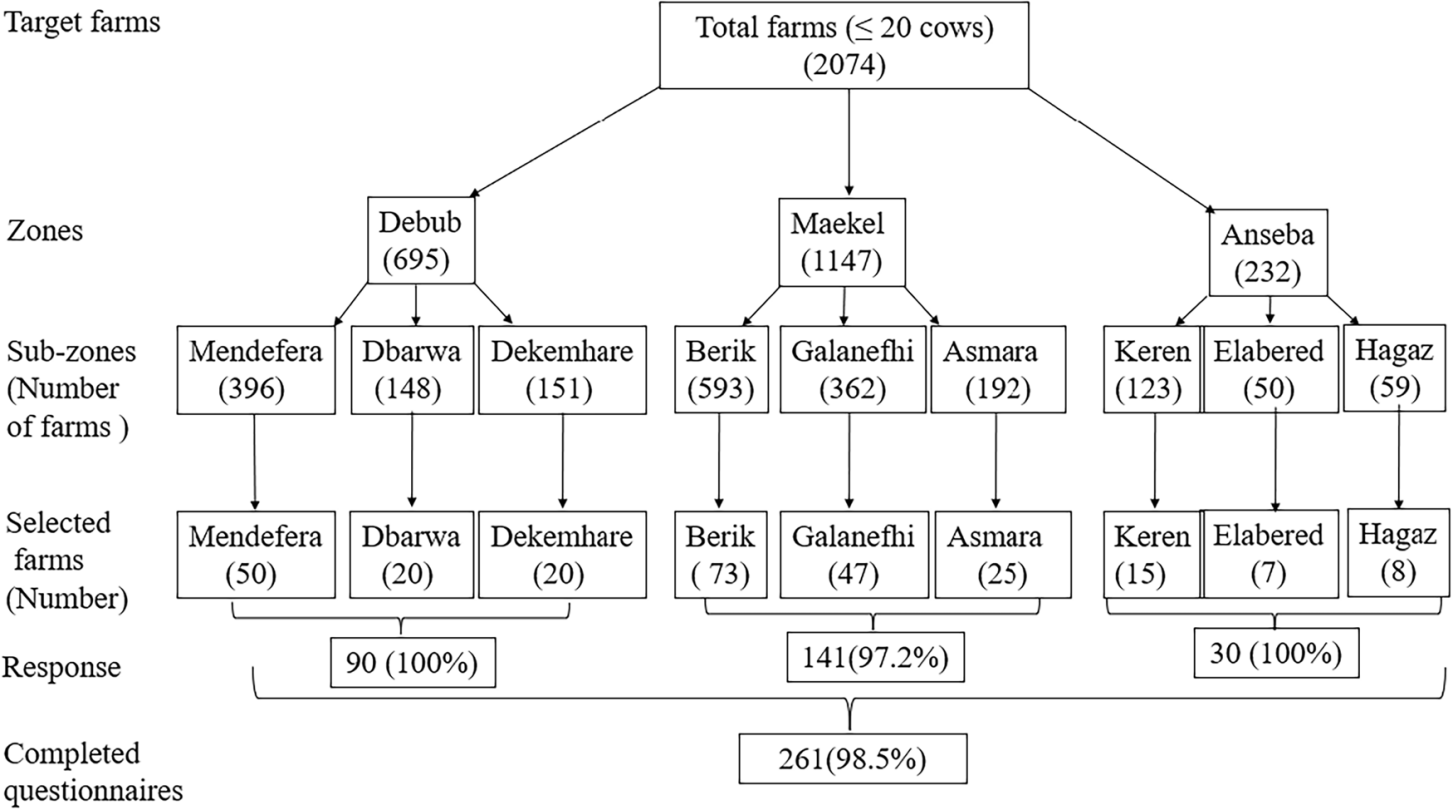
Illustration of sampling procedure

Primary data were collected from 261 smallholder dairy farmers using a multistage stratified random sampling method. This approach was chosen to ensure adequate representation of the major dairy-producing zones and to account for heterogeneity in production intensity and institutional access across zones. Stratification by zone and subzone was used to account for zonal differences in agroecological and institutional differences. Within each zone, three subzones were purposively selected based on intensity of dairy farming activities, and sample sizes were allocated proportionally according to the estimated number of dairy farmers in each subzone (Fig. 3).

Population estimates were obtained from the Ministry of Agriculture offices, and official lists of farms registered for milk production and sales served as the sampling frame for the random selection of individual respondents. These registries, maintained and periodically updated by local agricultural offices and verified by extension officers, provided a reliable and up-to-date source for sampling. For this study, the sampling frame consisted of 2074 farms with twenty or fewer cows to represent the smallholder segment of the dairy sector. As a result, the sampling frame did not include unregistered or informal producers who keep cattle for household consumption. The findings should therefore be interpreted as representative of the registered smallholder dairy sector rather than the wider rural livestock-keeping population.

Structured face-to-face interviews were conducted using a pre-tested questionnaire designed to capture farmers’ perceptions of climate change, socio-economic characteristics, and adaptation practices. To enhance data reliability and reduce communication barriers, the questionnaire was translated into the local language. In addition, six focus group discussions were held with farmers, and key informant interviews were conducted with dairy cooperative leaders and officials from the Ministry of Agriculture’s livestock units. These qualitative data provided contextual insights into local perceptions of climate change and institutional support mechanisms, enriching and triangulating the survey results.

### Sample Size Determination

The sample size was calculated using the finite population sample size formula outlined in Cochran (1977).

$${n}_{0}=\frac{{Z}^{2}p(1-p)}{{e}^{2}}$$

where Z = 1.96 corresponds to a 95% confidence level, *p* = 0.5 assuming maximum variability, and *e* = 0.05 is the desired precision level. For a finite population of *N* = 2074, the adjusted sample size is given by

$$n=\frac{{n}_{o}}{1+\frac{{n}_{0}-1}{N}}$$

Based on these parameters, the theoretical sample size needed was around 324 households. However, due to logistical considerations, a target sample of 265 dairy households was deemed sufficient. Out of the selected respondents, 261 participated in the interviews, resulting in a response rate of 98.5% (Fig. 3).

### Analytical Methods

#### Methods for Testing Validity and Reliability of Index Constructs

The reliability and validity of the index constructs were assessed through exploratory factor analysis and internal consistency checks. Factors were extracted using principal axis factoring, with retention guided by Horn’s parallel analysis with 1000 iterations (Xia & Zhou, 2025). Items with loadings ≥ 0.40 were retained, as recommended for the sample size (Field, 2018), and unidimensionality was confirmed when items loaded primarily on a single factor (Hair et al., 2019). Internal consistency was evaluated using Cronbach’s α ≥ 0.7 (Hussey et al., 2025) and corrected item–total correlations (≥0.30; Field, 2018), ensuring that the constructs were both reliable and conceptually coherent. Each index was normalized using the min–max scaling procedure, which rescales the predicted first factor scores to a standardized range between 0 and 1 for comparability across measures (Hair et al., 2019). The detailed results of the validity and reliability check for index construction are provided in Supplementary Materials (S2).

#### Methods for the Analysis of Factors Associated with Perception

This study adopted a parallel mediation analytical framework to examine how social, economic, and environmental variables relate to climate change perception, with threat appraisal and coping appraisal serving as mediating variables. This approach aligns with numerous previous studies that have applied the Protection Motivation Theory (PMT) and mediation frameworks to identify indirect associations. For example; Vrselja et al. (2024) found that risk judgments and level of worry about climate mediates the relationship between media exposure and pro-environmental behavior, while Chen (2020) reported that coping appraisal and moral obligation mediated the effect of threat appraisal on mitigation intentions, and Akoni et al. (2024) showed that protection motivation mediated the link between environmental awareness and drought-adaptation behaviors. Similarly, Hoffmann & Muttarak (2020) identified knowledge, awareness, and risk perception as mediators of the relationship between education and pro-environmental behavior. In other studies, Vrselja & Batini (2024) found that socioeconomic status operates through efficacy beliefs, and Yang et al. (2025) reported that risk perception mediates effects on environmentally responsible intentions.

Mediation analysis, as defined by Hayes (2022), quantifies the extent to which the effect of an independent variable on an outcome is transmitted through one or more mediators. Early methods, such as the causal steps framework proposed by Baron & Kenny (1986), laid the foundation for mediation analysis but relied heavily on significance testing and did not directly estimate indirect effects. To overcome these limitations, this study employs bootstrapping with 5000 resamples to generate bias-corrected confidence intervals for indirect effects. This approach provides more accurate estimates and aligns with current best practices in mediation analysis (Alfons et al., 2022; Hayes, 2022).

Specifically, this study employed a parallel mediation model, as outlined by Hayes (2022), within the Generalized Linear Model (GLM) framework using Jamovi (version 2.6.44) to examine factors associated with climate change perception. This regression-based approach allows the simultaneous assessment of two mediators: coping appraisal and threat appraisal in the relationships between socio-economic, institutional, and environmental variables and climate change perception, to examine both direct and indirect associations. The full (Eq. 1) and mediator models (Eqs. 2 and 3) are specified below.

1 $$\begin{array}{l}{Perception}={\beta }_{0}+{{\beta }_{1}{Coping}+{\beta }_{2}{Threat}}\\\qquad\qquad\qquad+\,{\beta }_{3}{Shade}+{\beta }_{4}{Extesion}+{\beta }_{5}{Media}+{\beta }_{6}{Education}\\+{\beta }_{7}{Heat}+{\beta }_{8}{Altitude}+{\beta }_{9}{Agexp}+{\beta }_{10}{Offarm}+{e}\end{array}$$

2 $$\begin{array}{l}{Coping}={\beta }_{0}+{\beta }_{1}{Shade}+{\beta }_{2}\\\qquad\qquad+\,{Extesion}+{\beta }_{3}{Media}+{\beta }_{4}{Education}+{\beta }_{5}{Heat}+{\beta }_{6}{Altitude}\\\qquad\qquad+{\beta }_{7}{Agexp}+{\beta }_{8}{Offarm}\ldots \ldots \ldots \ldots \ldots \ldots \ldots \ldots \ldots \ldots \ldots \ldots \ldots \ldots \ldots\end{array}$$

3 $$\begin{array}{l}{Threat}={\beta }_{0}+{\beta }_{1}{Shade}+{\beta }_{2}{Extesion}\\\qquad\qquad+{\beta }_{3}{Media}+{\beta }_{4}{Education}+{\beta }_{5}{Heat}+{\beta }_{6}{Altitude}\\\qquad\qquad+{\beta }_{7}{Agexp}+{\beta }_{8}{Offarm}\ldots \ldots \ldots \ldots \ldots \ldots \ldots \ldots \ldots \ldots \ldots \ldots \ldots \ldots \ldots\end{array}.$$

Where:${\beta }_{0}$ refers to the intercept in each equation, ${\beta }_{1}\ldots .{\beta }_{10}$ represents the effects of the independent variable on the dependent variable, and $e$ is the error term. The descriptions, scaling, and hypothesized effects of the variables are given in Table 3. Agexp refers to an interaction term of Age and Experience, included to examine how the relationship of accumulated farming experience with perception may depend on, or change with, a farmer’s age.

**Table 3:** Description of Variables and Hypothesized Effects on Farmers’ Climate Change Perception

Variable	Description	Apriori
Perception (Continuous)	The dependent variable, farmers’ perception of climate change, measured using an index constructed from four indicators outlined in the conceptual framework. Higher index values indicate a greater perception of climate change.	
Shade (Binary)	Indicates whether the farmer owns an improved dairy cattle shade different from their residential house. (1 = Yes, 0 = No).	Positive/ Negative
Experience (Continuous)	Total number of years engaged in dairy farming activities.	Positive
Age (Continuous)	Age of the respondent (in years).	Positive/Negative
Education (Ordinal)	Highest level of formal education attained: 1 = No formal education, 2 = Elementary, 3 = Junior, 4 = Secondary, 5= Vocational, 6 = University/college	Positive
Extension (Continuous)	A composite index constructed from five-point scale responses measuring farmers’ satisfaction with different types of extension services. Higher index values indicate greater satisfaction.	Positive
Media (Continuous)	A composite index for measuring media exposure constructed from farmers’ self-reported exposure to agricultural information programs through radio, television, magazines, and newspapers. Higher index values indicate greater media exposure.	Positive
Altitude (Continuous)	Elevation (in meters above sea level) of the farm’s location, measured by enumerators using mobile GPS applications.	Positive/Negative
Offarm (Binary)	Indicates receipt of income from off-farm sources, including remittances. (1 = Yes, 0 = No).	Negative
Heat (Binary)	Indicates whether the farmer has observed heat stress symptoms in cattle during the last five years (1 = Yes, 0 = No)	Positive
Threat	Threat appraisal: measured using an index of farmers perceived incidences of climatic risks, with higher scores indicating greater recognition of these risks.	Positive
Coping	Coping appraisal; measured using an index of previous adaptive practices as a proxy, with higher scores indicating greater coping appraisal.	Positive/negative

The direct, indirect, total, and full model effects were then examined to identify significant associations. An indirect effect was considered significant if the 95% confidence interval did not include zero (Hayes, 2022; Tibbe & Montoya, 2022), indicating that the mediator significantly transmitted the influence of the independent variable on climate change perception.

## Results

This section presents the results of the study. It begins with descriptive statistics of farmers’ socio-economic and farm characteristics, followed by findings on access to climate information, extension services, adaptation practices, and climate-related threats. Subsequently, the results of reliability and validity check of indices are presented. Finally, the results of the regression analysis, highlighting both direct and indirect associations among the study variables, are presented.

### Socio-economic Characteristics

Table 4 shows the descriptive statistics for selected socioeconomic characteristics of the respondents. On average, respondents were aged 52 years (SD = 13.76) and had 17.4 years of farming experience (SD = 10.86). The mean education level among respondents was 3.22 (SD = 1.19) on a scale from 1 (No formal education) to 6 (college/university). Approximately 64% of participants had attained primary or lower secondary education. The sample was predominantly male (82%), indicating a low level of female participation in dairy farms. These findings are consistent with the reports of Onakuse et al. (2025).

**Table 4:** Descriptive statistics of socio-economic characteristics

Variable	Obs.	Mean	Std. Dev.	Min	Max
Respondent’s age (years)	261	51.99	13.76	19	85
Years of farming experience	261	17.42	10.86	1	70
Education level of respondent (1–6 scale)	261	3.22	1.19	1	6
Gender of respondent (=1 if male)	261	0.82	0.39	-	-
Access to credit (=1 if yes)	260	0.19	0.40	-	-
Off-farm Income (=1 if yes)	261	0.09	0.29	-	-

Access to formal credit was limited, with only 19% of respondents using credit, while just 9.2% reported receiving off-farm income. Instead, farmers often depended on informal credit from relatives, friends, community associations, and wholesalers who purchased produce directly at the farm gate. In focus group discussions, farmers noted a preference for borrowing from buyers, as this arrangement provided both credit and a guaranteed market. However, it also compelled them to sell at prices below market value, underscoring the need for innovative financing mechanisms that broaden access to credit while safeguarding farmers’ bargaining power.

### Farm Characteristics

Looking at the descriptive statistics for farm characteristics (Table 5), only 44% of respondents had constructed a dedicated dairy cattle shade structure, and just 33% perceived their land tenure as secure, an important barrier to investing in long-term adaptive practices. In the focus group discussions, farmers had expressed their concerns about the lack of land for building dairy farms and forage production as a major constraint for dairy development.

**Table 5:** Descriptive statistics of farm characteristics

Variable	Obs.	Mean	Std. Dev.	Min	Max
Improved dairy cattle shade (=1 if yes)	258	0.44	0.50	-	-
Secure land tenure status (=1 if yes)	261	0.33	0.47	-	-
Farm altitude (meters above sea level)	260	2062	358	814	2443
Total landholding (hectares)	261	1.57	1.44	0.00	8.00
Dairy herd size (number of cows)	261	6.10	4.13	1	20
Heat stress observed (=1 if yes)	261	0.46	0.50	-	-

Farms were in the highlands and midlands at an average altitude of 2062 meters (SD = 358). These highland and midland regions are considered ideal for intensive dairy farming in Eritrea, where most registered dairy farms are located (Habtetsion, 2023). The average landholding per farm was 1.57 hectares (SD = 1.44), with an average dairy herd size of 6.1 cows (SD = 4.13). These figures indicate the smallholder nature of the sample. Most of these farms are mixed crop-livestock operations, with the majority of their land dedicated to grain production, primarily cereals (MoA, 2024).

Nearly half of the respondents (46%) reported seeing signs of heat stress in their animals. In discussions, farmers explained that they cope with these challenges by providing shade and extra water for their animals. A few also mentioned using water sprays on their livestock and adjusting feeding times to help mitigate the effects of heat.

### Climate Information and Extension Services

**Fig. 4:**
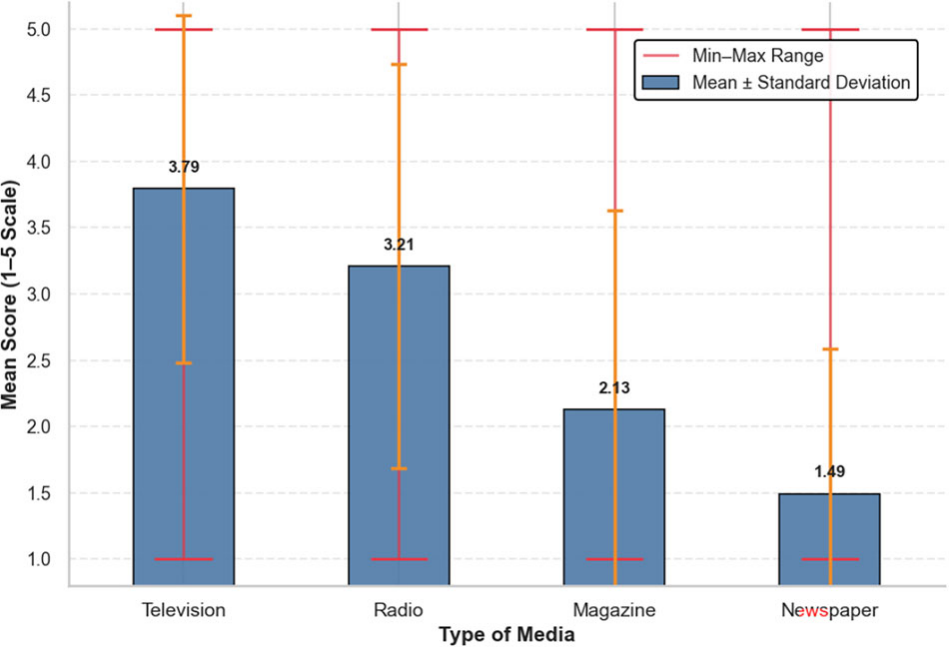
Media exposure summary statistics

Looking at respondents’ media exposure, varying levels of access to different media types were reported (Fig. 4). Radio and television were found to be the most frequently accessed sources, with mean scores of 3.21 and 3.79, respectively, on a 5-point scale. In contrast, print media such as magazines (*M* = 2.13) and newspapers (*M* = 1.49) were less commonly used. These findings suggest that electronic media, particularly television and radio, are the primary channels through which farmers obtain information.

These results were reinforced during group discussions, where farmers mentioned a radio program, *Hirshana Nemaebel* (“let’s develop our farming”), and a television program, *Fre-tsaeri* (“the fruit of hard work”), as their main sources of agricultural information. Ministry of Agriculture officers, in key informant interviews, likewise noted that these platforms are their primary means of reaching farmers nationwide, particularly for weather forecasts, locust alerts, and sharing success stories of exemplary farmers.

**Fig. 5:**
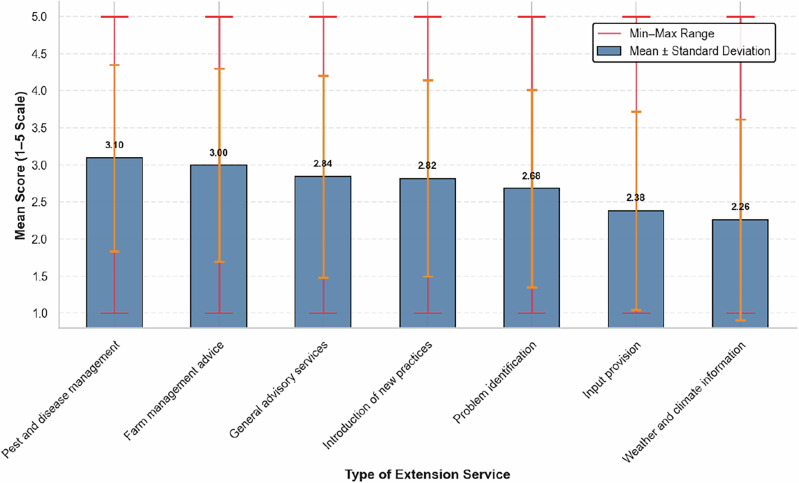
Descriptive statistics of farmers’ evaluation of extension services (1= Highly dissatisfied, 5= Highly satisfied)

Extension service in the study area is provided by the Ministry of Agriculture through extension agents working in collaboration with local administrations. Farmers participating in focus group discussions reported frequent contact with extension agents; however, their satisfaction varied across different areas of support (Fig. 5). The highest satisfaction was expressed with pest and disease control and farm management support, followed by advisory visits, demonstrations of new practices, and problem identification. In contrast, satisfaction was lowest for input provision and weather information, highlighting critical gaps in farmers’ access to timely inputs and reliable climate information. Farmers stressed that persistent input shortages compel them to rely on informal markets, where prices are inflated and quality often compromised. Extension agents, during key informant interviews, also acknowledged that the unavailability of farm inputs has negatively affected the effectiveness of their services.

### Farmers’ Perception of Climate Change

Farmers’ agreements with the proposed definition of climate change (Table 6), indicate the highest agreement for a gradual increase in temperature (*M* = 3.87, SD = 0.92), followed by changes in seasonal patterns (*M* = 3.39, SD = 0.92) and changes in rainfall patterns, including droughts and floods (*M* = 3.26, SD = 1.13). Moreover, the average agreement that rising greenhouse gas emissions are a cause of climate change was 2.43 (SD = 1.22).

**Table 6:** Descriptive statistics of farmers’ agreement level with proposed definitions of climate change (1= Strongly disagree, 5 = Strongly agree)

Definitions of climate change	Obs.	Mean	Std. Dev.	Min	Max
Changes in seasons (earlier/delayed planting)	261	3.391	0.916	1	5
Changes in rainfall patterns (droughts/floods)	260	3.258	1.125	1	5
Caused by a rise in greenhouse gases (e.g., CO₂)	255	2.431	1.221	1	5
A gradual increase in temperature	261	3.866	0.916	1	5

**Fig. 6:**
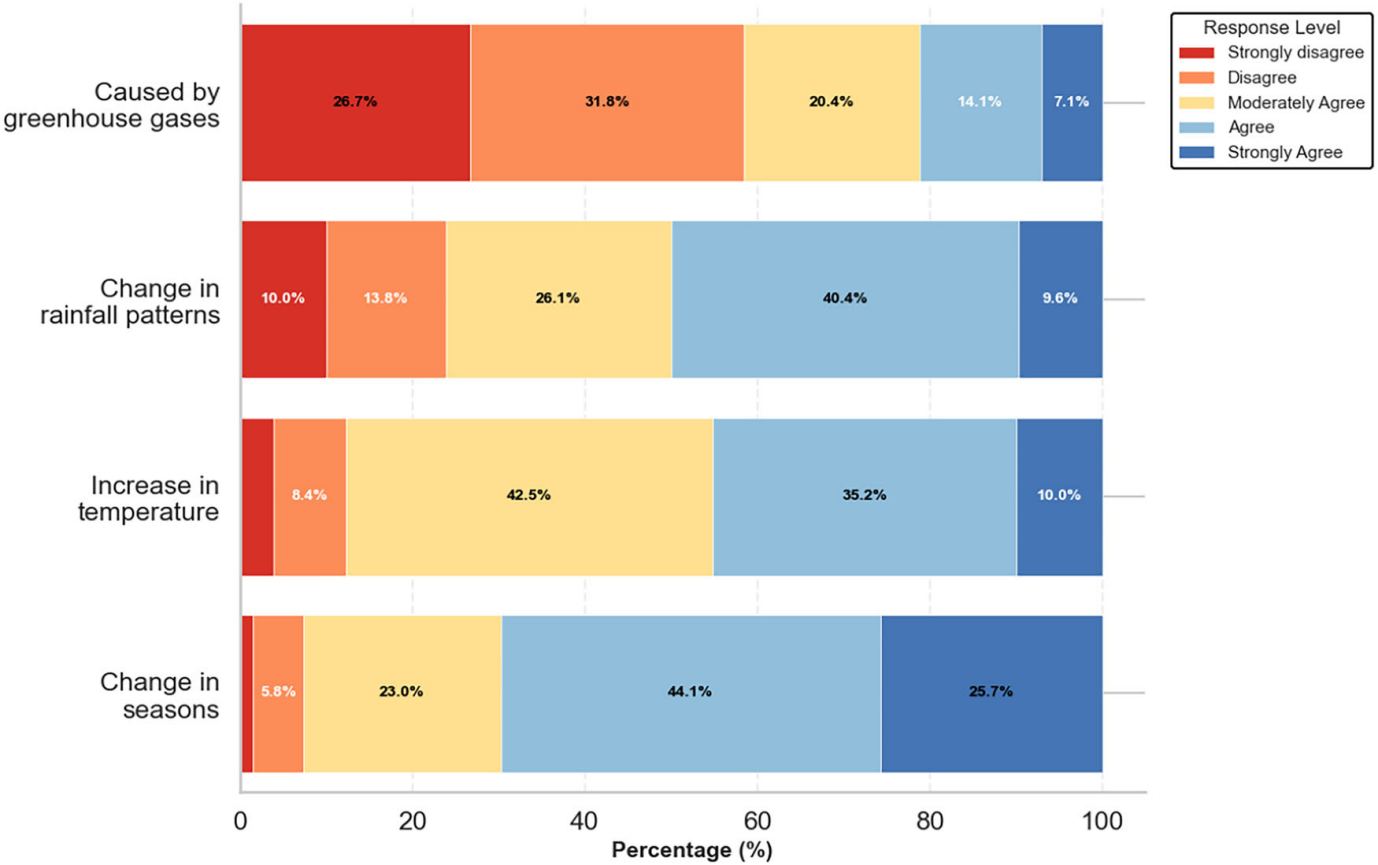
Percentage of farmers who agreed with the proposed definitions of climate change

The proportion of farmers who agree with these definitions presents a similar picture (Fig. 6). Overall, 93% of respondents linked climate change to shifting seasons, 76% to erratic rainfall, and 88% to rising temperatures. Furthermore, 41.6% identified greenhouse gas emissions as a cause. These findings point to a relationship between farmers’ perceptions and direct, observable environmental changes, rather than abstract or scientific concepts, consistent with prior research highlighting the salience of personal and localized experiences in climate change understanding (Armah et al., 2015; Khan et al., 2025; Ricart et al., 2023). This may also be explained by the limited scope of extension services, which often focus on delivering adaptation strategies without providing targeted climate education on the underlying causes and mechanisms of climate change.

### Previous Adaptive Practices

**Fig. 7:**
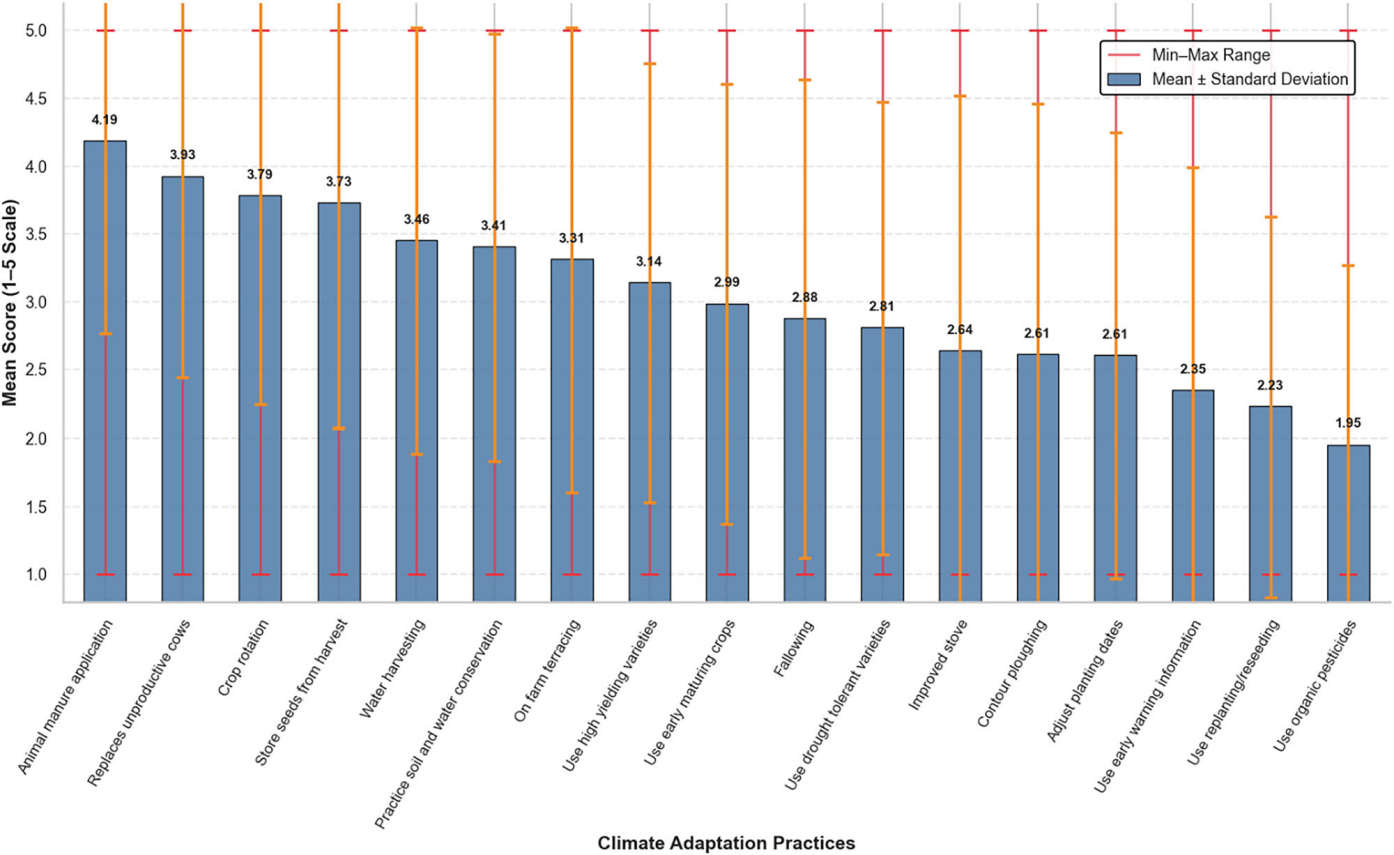
Descriptive statistics of climate adaptation practices (1 = Never, 5 = Always)

Farmers widely adopted various climate adaptation practices promoted by the Ministry of Agriculture through extension services (Fig. 7). Among the most common practices were the use of animal manure as a fertilizer (Mean = 4.19), replacement of unproductive cows (Mean = 3.93), crop rotation (Mean = 3.79), and seed storage (Mean = 3.73). While seed storage has long been a traditional practice among most smallholder farmers in Eritrea (FAO, 2022), the other measures have been strongly promoted by the Ministry of Agriculture (Ministry of Agriculture, 2020). This pattern highlights how adaptation strategies emerge from both generational knowledge and institutional influence, consistent with recent observations by (Mitter et al., 2024).

### Climate Change Threats

**Fig. 8:**
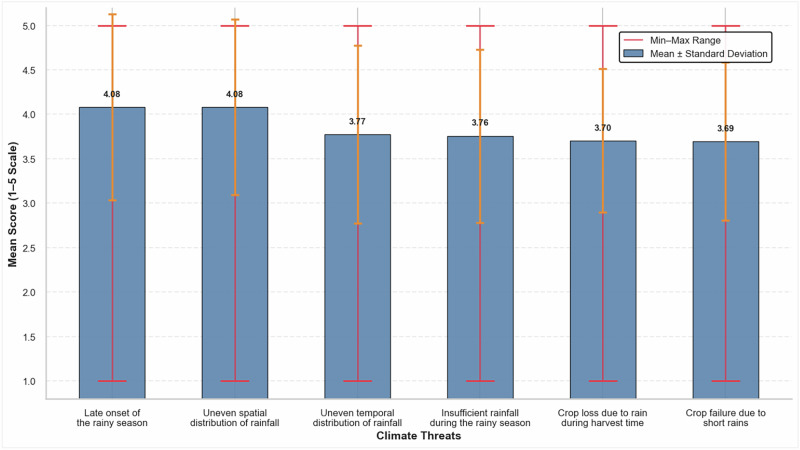
Descriptive statistics of climate-related threats (1= Never, 5 = Always)

Figure 8 presents the respondents’ perceived climate-related threats. The most frequently reported threats include late onset of rains (Mean = 3.70), uneven spatial rainfall distribution (Mean = 3.76), uneven temporal distribution (Mean = 3.77), and insufficient rainfall (Mean = 3.69). Respondents also identified crop losses resulting from short rains and untimely rainfall during harvest as particularly severe (Mean = 4.08), highlighting their critical impact on agricultural production. These findings align with broader East African evidence on climate risks (Doherty et al., 2022).

In sum, these findings indicate that farmers in the study area are operating in undeveloped input-output markets, with limited options for affordable credit and climate information. These findings imply the need for policy measures that strengthen essential support services for farmers. Priorities include improving input supply chains, expanding access to affordable credit, and providing timely, reliable weather and climate information. Aligning extension services with farmers’ practical needs and climate challenges can make agricultural programs more effective and help farmers make better-informed decisions in the face of climatic variability.

### Indices and Measures

The composite scores derived from factor analysis (Table 7) show farmers’ perceptions, threat and coping appraisals, and media exposure. The perception score (*M* = 0.569, SD = 0.196) reflects farmers’ overall awareness and understanding of climate change. The coping appraisal score (*M* = 0.529, SD = 0.279), measured as a proxy through farmers’ previous adaptive practices, indicates moderate but variable levels of coping appraisal. The threat appraisal score was the highest (*M* = 0.708, SD = 0.168), suggesting that farmers strongly recognize and rate climate-related risks as serious. Moreover, the average satisfaction with extension services was 0.436 (SD = 0.252), and media exposure averaged 0.366 (SD = 0.243), indicating limited access to institutional and media-based information sources. The distributions of these indices are provided in Supplementary Materials (S1).

**Table 7:** Descriptive Statistics of Index Constructs

Variable	Obs.	Mean	Std. Dev.	Min	Max
Perception score	255	0.569	0.196	0	1
Coping appraisal	259	0.529	0.279	0	1
Threat appraisal	252	0.708	0.168	0	1
Extension score	261	0.436	0.252	0	1
Media score	261	0.366	0.243	0	1

#### Reliability and Validity of Index Constructs

Each index was constructed using at least four items, in line with recommended practice for composite measures (Hair et al., 2019; van Valkengoed et al., 2021). Cronbach’s α values ranged from 0.67 to 0.91, and corrected item–total correlations from 0.35 to 0.78, indicating acceptable to excellent reliability across indices (Field, 2018; Hussey et al., 2025). As shown in Table 8, the first factor in each index had an eigenvalue greater than one. Horn’s parallel analysis further supported the retention of a single factor for each index. Factor loadings ranged from 0.40 to 0.92, all positive and within recommended thresholds (Osborne, 2005). Likelihood ratio tests were statistically significant (*p* < 0.001), confirming that items within each index were sufficiently related to support construct validity. The details of Cronbach’s alpha tests, factor loadings, and Horn’s parallel analysis are provided in supplementary material (S2).

**Table 8:** Reliability and Construct Validity of the Constructed Indices

Index	Cronbach’s α	Eigenvalue Factor 1	Eigenvalue Factor 2	Likelihood Ratio Test
Perception score	0.73	1.58	0.09	χ² (6) = 230.37, *p* < 0.001
Coping appraisal	0.92	7.44	0.85	χ² (136) = 2430.80, *p* < 0.001
Threat appraisal	0.74	2.06	0.23	χ² (15) = 349.09, *p* < 0.001
Extension	0.87	3.47	0.16	χ² (21) = 768.94, *p* < 0.001
Media exposure	0.67	1.31	0.11	χ² (6) = 173.47, *p* < 0.001

### Regression Results

This section presents the results of regression analysis used to examine the factors associated with climate change perception scores (Perception). Drawing on Protection Motivation Theory, this study hypothesized that threat appraisal (Threat) and coping appraisal (Coping) serve as pathways linking farm characteristics and information-related factors to farmers’ climate change perceptions. Independent variables including improved dairy shade (Shade), extension services (Extension), media exposure (Media), level of formal education (Education), entered as a squared term to capture potential non-linear effects, incidence of heat stress (Heat), log of altitude of the farm (Altitude), the interaction between age and farming experience (Agexp), and off-farm income (Offarm) are conceptualized as factors associated with appraisal mechanisms and climate change perception. All variables were measured simultaneously, and the mediation analysis was conducted to identify statistical relationships rather than temporal ordering.

Following this analytical framework, the study estimated a series of regression models to assess the direct effects, indirect effects, their components, total effects, and the corresponding individual pathways. The full model accounted for 30.6% of the variance in perception scores (F = 10.3, *p* < 0.001). Complete regression outputs, including diagnostics for multicollinearity and heteroskedasticity, are provided in Supplementary Materials (S3 and S4). All regression tables below report both unstandardized coefficients (Estimate) and standardized coefficients (β). For comparability, however, the interpretation of results, here and in the discussion, is based on the standardized coefficients. Significance for direct and total effects is evaluated using *p*-values, whereas indirect effects are assessed using bias-corrected bootstrapped confidence intervals.

#### Total Effects (X ⇒ Perception)

The regression results from the total effects model revealed that several variables were significantly associated with perception scores (Table 9). Extension services had the strongest positive association with perception (*β* = 0.355, *p* < 0.001), followed by media exposure (*β* = 0.210, *p* < 0.001) and education (*β* = 0.142, *p* = 0.014). In contrast, ownership of improved shade (*β* = –0.125, *p* = 0.027) and farm altitude (*β* = –0.187, *p* = 0.002) were both negatively associated with perception.

**Table 9:** Total effects (X ⇒ Perception)

			95% C.I.				
Names	Estimate	SE	Lower	Upper	*β*	*t*	*p*
Shade ⇒ Perception	−0.049	0.022	−0.095	−0.002	−0.125	−2.22	0.027
Extension ⇒ Perception	0.278	0.047	0.165	0.389	0.355	5.91	<0.001
Media ⇒ Perception	0.169	0.049	0.051	0.295	0.210	3.42	<0.001
Edu ⇒ Perception	0.003	0.001	0.001	0.006	0.142	2.45	0.014
Heat ⇒ Perception	0.032	0.022	−0.013	0.076	0.081	1.42	0.156
Altitude ⇒ Perception	−0.165	0.052	−0.274	−0.050	−0.187	−3.17	0.002
Agexp ⇒ Perception	0.002	0.001	0.000	0.005	0.082	1.45	0.146
Offarm ⇒ Perception	−0.068	0.038	−0.140	0.006	−0.100	−1.79	0.074

#### Mediator Models

The mediator models revealed significant associations between threat appraisal and multiple variables, whereas only extension services were associated with coping appraisal. As shown in Table 10, media exposure had a significant positive association with threat appraisal (*β* = 0.236, *p* < 0.001), suggesting that greater media engagement correlates with higher perceived climate-related threats. Similarly, heat stress (*β* = 0.147, *p* = 0.022) and the interaction between age and farming experience (*β* = 0.166, *p* = 0.008) were also positively associated with threat appraisal. In the coping appraisal model (Table 11), extension services exhibited a strong positive association (*β* = 0.441, *p* < 0.001), indicating that greater access to extension support is associated with higher levels of coping appraisal.

**Table 10:** Regression results for mediator model (X ⇒ Threat Appraisal)

			95% C.I.				
Names	Estimate	SE	Lower	Upper	*β*	*T*	*p*
Shade	−0.024	0.021	−0.066	0.017	−0.073	−1.164	0.246
Extension	0.045	0.044	−0.042	0.133	0.068	1.021	0.308
Media	0.163	0.047	0.069	0.256	0.236	3.436	<0.001
Education	0.002	0.001	−0.001	0.005	0.097	1.514	0.131
Heat	0.049	0.021	0.007	0.091	0.147	2.303	0.022
Altitude	−0.040	0.049	−0.136	0.057	-0.053	-0.810	0.419
Agexp	0.004	0.001	0.001	0.006	0.166	2.678	0.008
Offarm	0.007	0.036	−0.064	0.078	0.012	0.187	0.852

**Table 11:** Regression results for mediator model (X ⇒ Coping Appraisal)

			95% C.I.				
Names	Estimate	SE	Lower	Upper	*β*	*t*	*p*
Shade	−0.017	0.032	−0.079	0.046	−0.030	−0.526	0.599
Extension	0.489	0.067	0.356	0.622	0.441	7.258	<0.001
Media	0.118	0.071	−0.022	0.259	0.103	1.656	0.099
Education	0.004	0.002	0.000	0.008	0.103	1.753	0.081
Heat	−0.005	0.032	−0.069	0.059	−0.009	−0.151	0.880
Altitude	−0.034	0.075	−0.181	0.113	−0.027	−0.459	0.646
Agexp	0.000	0.002	−0.004	0.004	−0.008	−0.145	0.885
Offarm	0.054	0.055	−0.055	0.162	0.056	0.970	0.333

#### Direct Effects

Results from the full model demonstrated significant associations between the predictors and climate change perception scores (Table 12). Threat appraisal showed a positive and statistically significant association with perception (*β* = 0.184, *p* = 0.002), suggesting that farmers who perceive greater climate-related threat tend to show higher perception scores. Extension services also remained a strong positive association (*β* = 0.327, *p* < 0.001), and both media exposure (*β* = 0.157, *p* = 0.017) and formal education (*β* = 0.122, *p* = 0.043) were positively associated with perception.

**Table 12:** Direct effects (All Predictors ⇒ Perception)

			95% C.I.				
Name	Estimate	SE	Lower	Upper	*β*	*t*	*p*
Coping	0.033	0.045	−0.056	0.123	0.048	0.736	0.463
Threat	0.215	0.070	0.078	0.352	0.184	3.089	0.002
Shade	−0.044	0.023	−0.089	0.001	−0.113	−1.936	0.054
Extension	0.254	0.053	0.150	0.358	0.327	4.817	<0.001
Media	0.126	0.052	0.023	0.230	0.157	2.411	0.017
Education	0.003	0.001	0.000	0.006	0.122	2.038	0.043
Heat	0.020	0.023	−0.026	0.066	0.052	0.870	0.385
Altitude	−0.157	0.053	−0.261	-0.053	−0.180	−2.977	0.003
Agexp	0.001	0.001	−0.002	0.004	0.045	0.774	0.440
Offarm	−0.072	0.039	−0.149	0.004	−0.107	−1.875	0.062

Moreover, altitude showed a significant negative association with perception (*β* = –0.180, *p* = 0.003), indicating that farmers in higher-elevation areas tend to report lower perception levels. Ownership of improved dairy shade showed a marginal negative relationship (*β* = –0.113, *p* = 0.054), and off-farm income also demonstrated a marginal negative association (*β* = –0.107, *p* = 0.062). Other variables, including coping appraisal (*β* = 0.048, *p* = 0.463), heat stress (*β* = 0.052, *p* = 0.385), and the age–experience interaction term (Agexp) (*β* = 0.045, *p* = 0.440) did not show significant associations in the full model.

#### Indirect Effects

The analysis of indirect effects revealed significant indirect associations through threat appraisal, whereas no significant indirect pathways were found through coping appraisal. For brevity, only the results for the threat appraisal pathway are presented here, while full results are provided in the supplementary material (S3). As shown in Table 13, media exposure demonstrated a significant indirect association with climate change perception (*β* = 0.046, 95% CI [0.01194, 0.08025]). The combined age–experience variable also revealed a significant indirect association (*β* = 0.034, 95% CI [0.0002, 0.0018]). In addition, observed heat stress exhibited a significant indirect association with perception (*β* = 0.023, 95% CI [0.0001, 0.0253]).

**Table 13:** Indirect effects via threat appraisal

			95% C.I. (a)				
Effect	Estimate	SE	Lower	Upper	*β*	*z*	*p*
Shade ⇒ Threat ⇒ Perception	−0.01	0.01	−0.02	0.00	−0.01	−1.13	0.26
Extension ⇒ Threat ⇒ Perception	0.01	0.01	0.00	0.04	0.02	1.19	0.23
Media ⇒ Threat ⇒ Perception	0.04	0.02	0.01	0.08	0.05	2.41	0.02
Education ⇒ Threat ⇒ Perception	0.00	0.00	0.00	0.00	0.02	1.54	0.12
Heat ⇒ Threat ⇒ Perception	0.01	0.01	0.00	0.03	0.02	1.69	0.09
Altitude ⇒ Threat ⇒ Perception	−0.01	0.01	-0.04	0.02	−0.01	−0.73	0.46
Agexp ⇒ Threat ⇒ Perception	0.00	0.00	0.00	0.00	0.03	2.16	0.03
Offarm ⇒ Threat ⇒ Perception	0.00	0.01	−0.01	0.02	0.00	0.15	0.88

Confidence intervals were estimated with bias-corrected bootstraps, and betas are completely standardized effect sizes.

#### a. Sensitivity Analysis of Indirect Effects

**Fig. 9:**
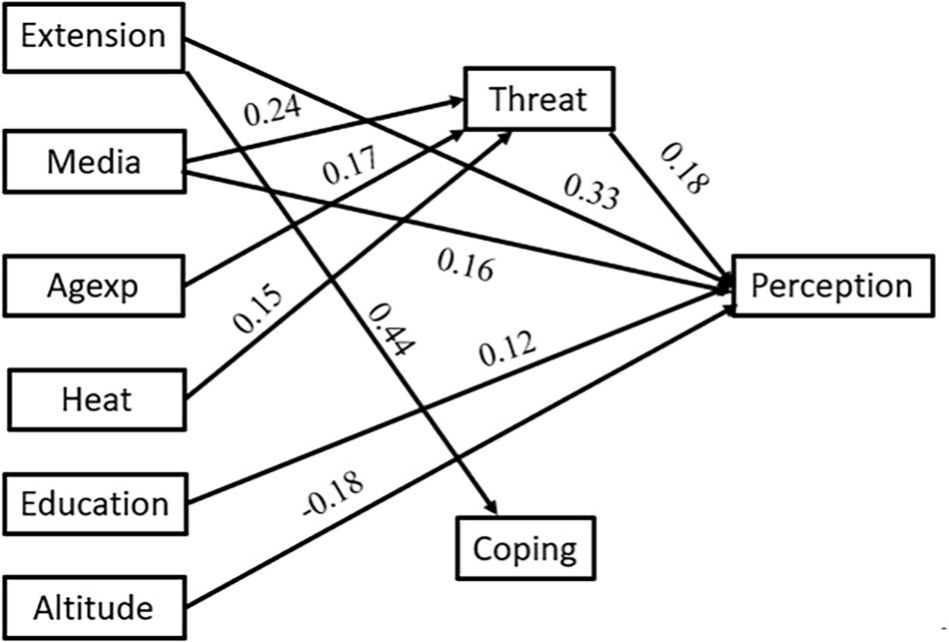
Path diagram of direct and indirect associations (only significant, *p* < 0.05, paths are shown)

Sensitivity analyses were conducted to evaluate the robustness of the mediation effects to potential unmeasured confounding. For all exposure variables, both the specific indirect effects through Threat and the total indirect effects remained statistically significant (*p* < 0.05) across the full range of tested sensitivity parameters (*ρ* = ±0.8). Thus, despite the small sizes of these indirect effects, only substantial unmeasured confounding (*ρ* ≥ |0.8 | ) would be sufficient to eliminate the observed mediation effects. Complete sensitivity analysis results are provided in Supplementary Materials (S5), with direct and indirect pathways illustrated in Fig. 9.

## Discussion

This study aimed to (1) develop and validate indices of smallholder farmers’ threat appraisal, coping appraisal, and climate-change perception, and (2) assess how socioeconomic, institutional, and environmental factors relate to climate-change perceptions, including indirect effects through appraisal mechanisms. The following section discusses the above-stated findings.

The average normalized score for farmers’ threat appraisal was 0.71, compared with 0.53 for coping appraisal and 0.57 for overall climate-change perception. These figures indicate that smallholder dairy farmers in Eritrea recognize climate change as a serious threat to their livelihoods. The moderate overall perception score is attributed to the lower score on the question assessing understanding of the underlying causes of climate change, despite relatively stronger recognition of its observable manifestations and consequences (e.g., rising temperatures, extreme rainfall events, shifting seasonality). This pattern is consistent with findings from similar studies across eastern Africa (Debela et al., 2015; Kahsay et al., 2019; Nyang’au et al., 2021).

The limited understanding of greenhouse gases as a cause (41.6%) may also partly explain the relatively low coping appraisal score. In a situation where farmers perceive the threats with a low understanding of the underlying causes, they may attribute them to natural phenomena or external forces rather than human-driven processes. As a result, adaptation responses often rely on external supports such as project-based interventions or community initiatives, rather than individual agency. For example, Debela et al. (2015) reported that 45% of smallholder farmers in southern Ethiopia attribute climate change to supernatural processes. The limited individual adaptation practices in the study area could be due to lack of resources, information and inadequate institutional support.

The regression-based mediation analysis showed a significant positive association between threat appraisal and climate change perception (18.4%, *p* < 0.01), indicating that farmers who recognize climate-related risks tend to have higher levels of climate change perception. Similar associations have been documented in Ethiopia (Etana et al., 2021), Kenya (Rao et al., 2011), and France and Australia (Bradley et al., 2020).

Extension services (35.5%, *p* < 0.01) and media exposure (21%, *p* < 0.01) were also positively associated with perception, both directly and indirectly, through threat appraisal. These findings align with evidence that agricultural extension and media serve as important communication channels associated with farmers’ understanding of climate risks. Studies from Iran (Azadi et al., 2019), South Africa (Makamane et al., 2025), Zimbabwe (Zamasiya et al., 2017), and Ethiopia (Zeleke et al., 2023) similarly show that information and advisory services are closely linked with increased awareness and interpretation of climate-related changes.

The association between media exposure and farmers’ climate-change perceptions in this study is particularly noteworthy. Media coverage often highlights vivid, event-based climate impacts rather than the underlying scientific mechanisms (Boykoff & Rajan, 2007), and global comparative analyses show that news outlets across regions disproportionately emphasize impacts and extreme events over causal drivers (Keller et al., 2021). Such framing can reinforce manifestation-based beliefs while doing little to strengthen causal understanding. In agricultural settings, where both extension services and media serve as primary information sources, this tendency may further amplify farmers’ attention to observable climate signals while leaving causal attributions comparatively underdeveloped.

Education was also positively associated with climate change perception (14.2%, *p* < 0.05). This pattern is consistent with evidence from Sub-Saharan Africa showing that educational attainment supports farmers’ ability to interpret climate information and attribute observed changes to climate drivers (Ndlovu & Ndlovu, 2025; Tshabalala et al., 2025).

Further, the mediation results indicate that threat appraisal plays a vital role in some of the observed associations. Media exposure was partially mediated by threat appraisal. Thus, its association with perception was significant and strengthened among farmers who appraised climate risks as severe and personally relevant. In other words, media engagement was associated with higher perceptions of climate change, and this association was partly explained by increased threat appraisal. In line with this finding Vrselja et al. (2024) reported the mediating role of cognitive risk judgment and worry in the association between media exposure and pro-environmental behavior. Moreover, similar results were reported by Azadi et al. (2019) who identified risk salience as an important correlate of adaptation-related judgments, and by Han et al. (2022), who noted that threat appraisal is closely related to adaptive responses. Additionally, the interaction of age and farming experience was found to be fully mediated by threat appraisal. This shows that older farmers, particularly those with more farming experience, exhibit higher perception levels when climate risks are internally appraised as consequential. These findings align with PMT’s proposition and recent reports that show risk judgements and interpretation of risk signals mediate the relationships of socioeconomic variables and pro-environmental behavior (Maddux & Rogers, 1983; Vrselja & Batini, 2024; Yang et al., 2025).

Environmental and livelihood factors also demonstrated significant associations. Farmers in higher-altitude areas reported lower levels of concern than those in lower-altitude areas (–18.7%, *p* < 0.01). This may be because farmers in the higher, moist highland agroecological zones perceive themselves as less exposed to climate-related risks compared to farmers in lower-altitude zones, where climate variability is typically more pronounced. This is further supported by the significant association between observed heat stress and threat appraisal. Although the association between heat stress and perception was fully mediated by threat appraisal, heat stress itself showed a significant positive association (14.7%, *p* < 0.05) with threat appraisal. These findings are consistent with research from Ethiopia and Iran indicating that reduced exposure to climatic extremes is often associated with lower perceptions of climate risk (Gemeda et al., 2023; Moridi et al., 2025).

Similarly, ownership of shade structures showed a negative association with lower perception scores (−12.5%, *p* < 0.05). Within the PMT framework, such association may reflect stronger coping appraisal but weaker threat appraisal, as assets can reduce perceived vulnerability. Comparable associations have been documented in Ghana (Ndamani & Watanabe, 2017), Pakistan (Jabbar et al., 2020), and Burkina Faso (Kannan et al., 2022), where greater asset ownership or wealth is linked with reduced perceived exposure to climatic threats.

Although coping appraisal was not a significant mediator in this study, previous studies have highlighted its importance as a factor associated with climate change perception and adaptive behavior (Grothmann & Patt, 2005; Mitter et al., 2019; Pakmehr et al., 2021). In this study, coping appraisal was proxied by farmers’ previous adaptive practices, which may partly explain its non-significant association. While studies elsewhere report strong links between adaptive practices and self-efficacy and response efficacy (Doran et al., 2020; van Valkengoed & Steg, 2019), such links may weaken in resource-constrained settings where adaptation depends heavily on project support or collective arrangements rather than individual agency (Chinseu et al., 2019). The finding that extension access was positively associated (44%, *p* < 0.01) with coping appraisal, yet coping appraisal itself was not linked to climate-change perception, may also be explained by the nature of extension services and resource constraints in the study area. In many low-income agricultural settings, extension services focus on practical, technical advice, rather than on explicitly teaching the underlying causes of climate change (Debela et al., 2015; Mitter et al., 2019). Consequently, farmers may acquire skills and experience in adaptation (reflected in coping appraisal) without this translating into deeper climate literacy or understanding of causality.

## Conclusions and Policy Implications

This study contributes to the limited empirical evidence on smallholder farmers’ perceptions of climate change in Eritrea. By drawing on a multidimensional perception index and examining socioeconomic, institutional, environmental, and psychological correlates, the results show a moderate overall level of perception (average normalized score: 0.57). Most farmers associated climate change with shifting seasons (93%), erratic rainfall (76%), and rising temperatures (88%). Moreover, only 41.6% identified greenhouse gas emissions as a defining factor, indicating that perceptions are primarily grounded in observable local environmental changes rather than scientific drivers. This suggests that while farmers are aware of climate variability, their understanding of its causes remains limited.

Extension services, media exposure, and formal education were positively associated with climate change perception, underscoring the role of access to information and knowledge in shaping awareness. Farmers in higher-altitude areas and those owning improved shade structures tend to have lower perception scores. The mediation analysis further showed that threat appraisal partially explained the associations between media exposure and climate change perception, as well as the association involving the interaction of age and experience. Coping appraisal, however, did not show a significant relationship with perception in this context.

These findings have several policy implications. First, improving farmers’ access to climate information through extension and media channels is crucial to strengthening climate change perception. Extension programs could pilot climate-literacy modules within regular visits, integrating locally relevant examples and short pre- and post-training assessments to measure changes in perception or threat-appraisal scores. Likewise, radio and community-based communication campaigns could be designed using behavioral insights from threat-appraisal theory, emphasizing perceived risks and adaptive efficacy, and evaluated through periodic perception index surveys.

Second, the positive association between education and perception highlights the importance of farmer-centered learning initiatives that embed climate content in both formal and non-formal agricultural training. These initiatives can be evaluated through participatory evaluation designs that track shifts in farmers’ interpretation of climate information over time.

Finally, tailoring climate messages to local conditions, such as altitude, temperature variation, or exposure to specific climatic risks, can enhance message relevance and comprehension. Linking these actions to measurable outcomes, such as improvements in the climate perception index or subcomponents of threat and coping appraisal, would allow for systematic learning and policy refinement. Overall, strengthening climate communication and advisory systems, anchored in testable, evidence-based interventions, would improve climate literacy and foster informed decision-making among smallholder farmers. These approaches can contribute to building more resilient agricultural livelihoods in climate-vulnerable settings such as Eritrea.

## Study Limitations

This study has several limitations that should be considered when interpreting the findings. First, the use of cross-sectional data limits causal inference. The results of the regression analysis should be understood as associations rather than causal mechanisms. Second, the regression-based mediation framework assumes that there are no unmeasured confounders affecting either the mediator or the outcome, but this assumption may only be partially met. While bootstrapped indirect effects quantify sampling uncertainty, they do not eliminate potential bias from omitted variables or correlated errors. Third, measurement limitations may have influenced the results. Coping appraisal was proxied by previous adaptation practices, which may not fully capture psychological constructs such as self-efficacy or response efficacy. Additionally, self-reported indicators of extension contact and media exposure may be subject to recall or social desirability bias. Future studies using longitudinal or quasi-experimental designs, along with more comprehensive measures of psychological constructs, would strengthen evidence on the pathways linking information, risk appraisal, and climate change perception.

## Supplementary information

README

Supplementary Materials 1_5

Synthetic_data

## Data Availability

The data supporting the findings of this study are not publicly available for privacy and ethical reasons, but can be accessed from the corresponding author upon reasonable request and with approval from the relevant ethics committee.
