# Supplementary material for: Factors Affecting Smallholders’ Perception of Climate Change in Eritrea
Source: Environ Manage. 2026 Jan 30;76(3):90. doi: 10.1007/s00267-026-02383-7 (PMC12858499; doi:10.1007/s00267-026-02383-7)
Supplement: Supplementary file 1 — README [file 267_2026_2383_MOESM1_ESM.pdf]

Supplementary Material (S1): Density Plots of Index Constructs

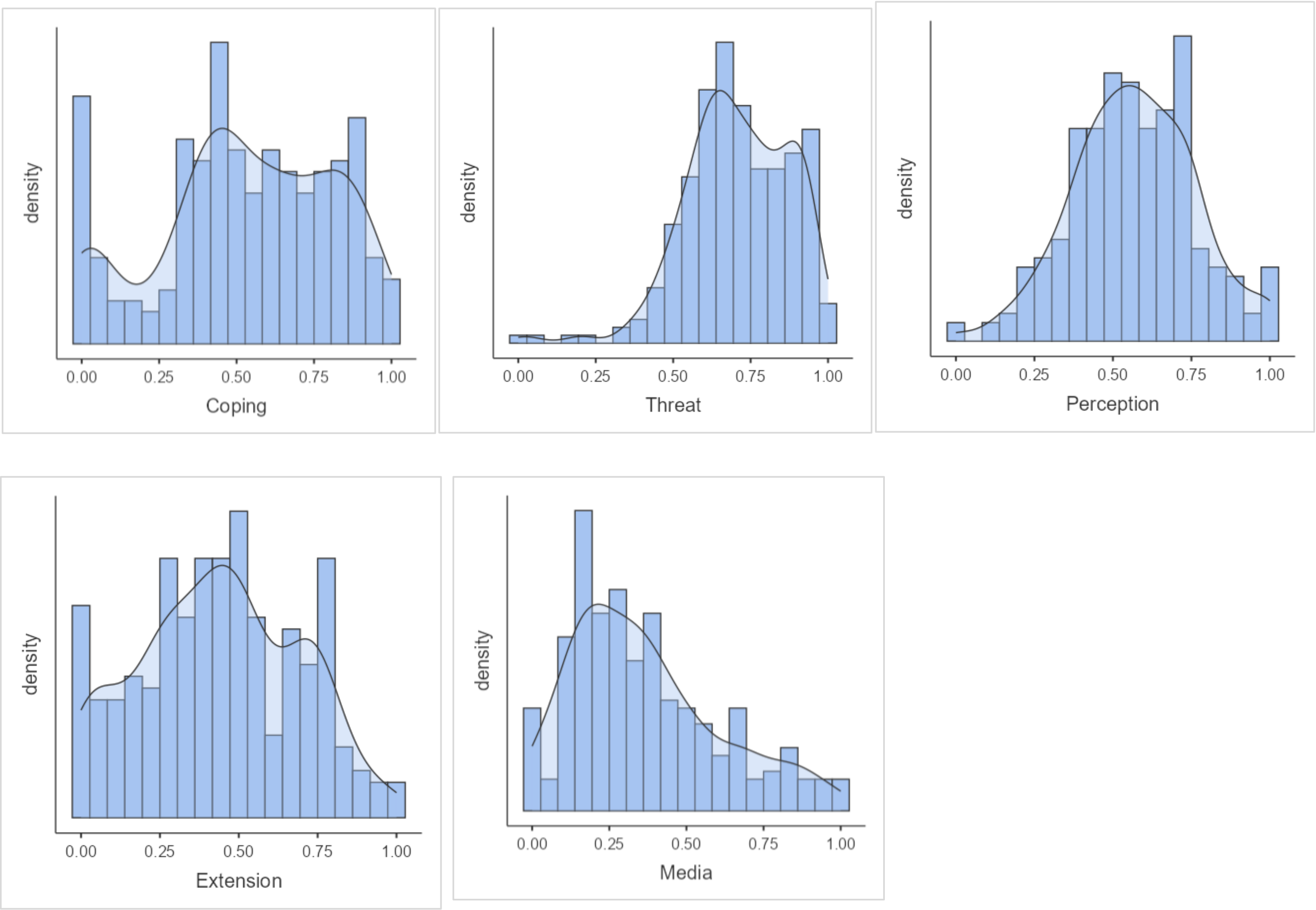

Supplementary Material (S2): Cronbach's Alpha Tests, Factor Analysis, and Horn's Parallel Analysis

i. Cronbach's alpha tests

```
. alpha Temprature Rainpattern GHgas Seasonchange ,item

Test scale = mean(unstandardized items)
```

Item	Obs	Sign	item-test correlation	item-rest correlation	average interitem covariance	alpha
Temprature	261	+	0.7792	0.6133	.4297668	0.6272
Rainpattern	260	+	0.7932	0.5804	.3879329	0.6364
GHgas	255	+	0.7597	0.4960	.4227869	0.6944
Seasonchange	261	+	0.6530	0.4293	.5547893	0.7219
Test scale					.4487067	0.7319

```
. alpha late_rain unevenspace_rain uneventemp_rain Insuff_rain losshar vest_rain cropfail_rain ,item

Test scale = mean(unstandardized items)
```

Item	Obs	Sign	item-test correlation	item-rest correlation	average interitem covariance	alpha
late_rain	259	+	0.6787	0.5329	.2987532	0.6929
unevenspac~n	258	+	0.7080	0.5369	.2734766	0.6853
uneventemp~n	256	+	0.7708	0.6204	.2488152	0.6595
Insuff_rain	258	+	0.5366	0.3337	.3384771	0.7400
lossharves~n	257	+	0.5886	0.3564	.3162416	0.7402
cropfail_r~n	258	+	0.6838	0.4993	.2816617	0.6960
Test scale					.2928894	0.7404

```
. alpha waterharvesting onfarm_terrace contour_ploughing fallowing crop_rotation animal_manure improved_stove early_mature high_yielding replace_
> unproduct organic_pesticide store_seeds replanting Earling_warning adjusting_plant soil_conservation tolerant_varities ,item

Test scale = mean(unstandardized items)
```

Item	Obs	Sign	item-test correlation	item-rest correlation	average interitem covariance	alpha
waterharve~g	261	+	0.7137	0.6678	1.067517	0.9172
onfarm_ter~e	261	+	0.7567	0.7124	1.044586	0.9158
contour_pl~g	261	+	0.5443	0.4674	1.092518	0.9230
fallowing	259	+	0.7196	0.6682	1.050539	0.9171
crop_rotat~n	261	+	0.7635	0.7248	1.057704	0.9158
animal_man~e	261	+	0.6980	0.6548	1.083075	0.9177
improved_s~e	261	+	0.5364	0.4574	1.093343	0.9235
early_mature	261	+	0.8007	0.7655	1.042015	0.9145
high_yield~g	261	+	0.8144	0.7813	1.038639	0.9141
replace_un~t	261	+	0.5601	0.5007	1.109095	0.9213
organic_pe~e	261	+	0.5168	0.4612	1.128298	0.9220
store_seeds	261	+	0.7360	0.6901	1.054215	0.9165
replanting	261	+	0.4617	0.3986	1.135429	0.9235
Earling_wa~g	261	+	0.6610	0.6061	1.074901	0.9187
adjusting_~t	261	+	0.6063	0.5449	1.088124	0.9203
soil_conse~n	261	+	0.7905	0.7550	1.048869	0.9149
tolerant_v~s	261	+	0.7183	0.6697	1.05805	0.9170
Test scale					1.074523	0.9229

```
. alpha problem_identify farm_manage pest_disease weather_info new_practice input_provision general_advise ,item

Test scale = mean(unstandardized items)
```

Item	Obs	Sign	item-test correlation	item-rest correlation	average interitem covariance	alpha
problem_id~y	261	+	0.7980	0.7098	.8452628	0.8490
farm_manage	261	+	0.7961	0.7099	.8526152	0.8492
pest_disease	261	+	0.7738	0.6842	.8748207	0.8529
weather_info	261	+	0.7350	0.6241	.8812241	0.8605
new_practice	261	+	0.7340	0.6258	.8868651	0.8602
input_prov~n	261	+	0.7447	0.6389	.8786227	0.8585
general_ad~e	261	+	0.7097	0.5904	.8963326	0.8650
Test scale					.8736776	0.8745

```
. alpha Radio Television Magazine Newspaper ,item

Test scale = mean(unstandardized items)
```

Item	Obs	Sign	item-test correlation	item-rest correlation	average interitem covariance	alpha
Radio	261	+	0.7232	0.4339	.6220601	0.6282
Television	261	+	0.6808	0.4253	.693015	0.6279
Magazine	261	+	0.7773	0.5270	.5152373	0.5579
Newspaper	261	+	0.6696	0.4645	.7272473	0.6136
Test scale					.6393899	0.6745

ii. Factor Analysis

```
. factor Temprature Rainpattern GHgas Seasonchange
(obs=255)

Factor analysis/correlation                        Number of obs    =        255
    Method: principal factors                      Retained factors =          2
    Rotation: (unrotated)                          Number of params =          6
```

Factor	Eigenvalue	Difference	Proportion	Cumulative
Factor1	1.57977	1.48048	1.2197	1.2197
Factor2	0.09929	0.24783	0.0767	1.2964
Factor3	-0.14854	0.08676	-0.1147	1.1817
Factor4	-0.23530	.	-0.1817	1.0000

```
    LR test: independent vs. saturated:  chi2(6)  =  230.37 Prob>chi2 = 0.0000

Factor loadings (pattern matrix) and unique variances
```

Variable	Factor1	Factor2	Uniqueness
Temprature	0.7081	0.0856	0.4913
Rainpattern	0.6599	-0.0974	0.5550
GHgas	0.5912	-0.1906	0.6141
Seasonchange	0.5416	0.2149	0.6605

```
. factor late_rain unevenspace_rain uneventemp_rain Insuff_rain lossharvest_rain cropfail_rain
(obs=252)

Factor analysis/correlation                        Number of obs    =        252
    Method: principal factors                      Retained factors =          3
    Rotation: (unrotated)                          Number of params =         15
```

Factor	Eigenvalue	Difference	Proportion	Cumulative
Factor1	2.05953	1.82674	1.1102	1.1102
Factor2	0.23280	0.16351	0.1255	1.2357
Factor3	0.06929	0.19138	0.0374	1.2731
Factor4	-0.12209	0.06469	-0.0658	1.2073
Factor5	-0.18678	0.01092	-0.1007	1.1066
Factor6	-0.19770	.	-0.1066	1.0000

```
    LR test: independent vs. saturated:  chi2(15) =  349.09 Prob>chi2 = 0.0000

Factor loadings (pattern matrix) and unique variances
```

Variable	Factor1	Factor2	Factor3	Uniqueness
late_rain	0.6120	0.0514	0.1275	0.6065
unevenspac~n	0.6774	-0.2548	-0.0570	0.4729
uneventemp~n	0.7609	-0.1927	0.0016	0.3839
Insuff_rain	0.3899	0.1661	0.1450	0.7993
lossharves~n	0.4189	0.1978	-0.1613	0.7594
cropfail_r~n	0.5653	0.2478	-0.0524	0.6163

```
. factor waterharvesting onfarm_terrace contour_ploughing fallowing crop_rotation animal_manure improved_stove early_mature high_yielding replace
> _unproduct organic_pesticide store_seeds replanting Earling_warning adjusting_plant soil_conservation tolerant_varities
(obs=259)

Factor analysis/correlation                    Number of obs    =        259
    Method: principal factors                  Retained factors =          9
    Rotation: (unrotated)                      Number of params =        117
```

Factor	Eigenvalue	Difference	Proportion	Cumulative
Factor1	7.44534	6.59285	0.8224	0.8224
Factor2	0.85249	0.28942	0.0942	0.9166
Factor3	0.56308	0.13706	0.0622	0.9788
Factor4	0.42602	0.12160	0.0471	1.0258
Factor5	0.30441	0.10242	0.0336	1.0595
Factor6	0.20199	0.03048	0.0223	1.0818
Factor7	0.17152	0.07976	0.0189	1.1007
Factor8	0.09176	0.08996	0.0101	1.1109
Factor9	0.00180	0.02009	0.0002	1.1111
Factor10	-0.01829	0.03914	-0.0020	1.1090
Factor11	-0.05743	0.03443	-0.0063	1.1027
Factor12	-0.09186	0.01562	-0.0101	1.0926
Factor13	-0.10748	0.02922	-0.0119	1.0807
Factor14	-0.13670	0.02204	-0.0151	1.0656
Factor15	-0.15874	0.02142	-0.0175	1.0480
Factor16	-0.18016	0.07467	-0.0199	1.0281
Factor17	-0.25482	.	-0.0281	1.0000

LR test: independent vs. saturated: chi2(136) = 2430.80 Prob>chi2 = 0.0000

Factor loadings (pattern matrix) and unique variances

Variable	Factor1	Factor2	Factor3	Factor4	Factor5	Factor6	Factor7	Factor8	Factor9	Uniqueness
waterharve~g	0.6959	-0.1367	0.0323	0.0375	-0.3138	0.0351	0.0871	0.0271	0.0130	0.3864
onfarm_ter~e	0.7438	-0.4054	0.0315	-0.1141	0.0233	0.0669	0.0800	0.0318	-0.0031	0.2560
contour_pl~g	0.4952	-0.1912	0.0228	0.2090	0.2519	-0.0165	0.1881	-0.0001	0.0026	0.5749
fallowing	0.6975	-0.2301	0.0809	-0.2877	0.1216	0.0255	0.0280	0.0290	-0.0111	0.3541
crop_rotat~n	0.7624	0.0676	-0.3593	-0.0749	0.0313	0.0350	-0.0340	-0.0740	0.0044	0.2706
animal_man~e	0.7000	-0.1552	-0.3648	0.0405	0.0265	0.0651	-0.0735	-0.1017	0.0038	0.3306
improved_s~e	0.4734	-0.2316	0.1528	-0.2392	0.0445	-0.1181	-0.0389	-0.0034	0.0222	0.6237
early_mature	0.7916	0.1355	0.2156	0.0378	0.1099	0.0783	-0.0988	0.0603	-0.0048	0.2755
high_yield~g	0.8160	0.0343	0.1435	0.1261	0.1064	0.0340	-0.1934	0.0547	-0.0053	0.2436
replace_un~t	0.5257	-0.1366	0.2201	0.3000	-0.0751	-0.0947	-0.1186	-0.0829	0.0071	0.5310
organic_pe~e	0.4812	0.1237	0.0895	-0.0867	-0.1141	0.2417	-0.0691	-0.0110	0.0076	0.6612
store_seeds	0.7333	-0.0356	-0.2749	0.0718	-0.0072	-0.2209	-0.0738	0.0645	-0.0071	0.3217
replanting	0.4207	0.2439	-0.0837	0.2215	0.1213	0.0930	0.1291	0.0913	0.0160	0.6588
Earling_wa~g	0.6271	0.2725	0.1884	-0.0844	-0.0278	-0.1971	0.0847	-0.0583	0.0036	0.4397
adjusting_~t	0.5922	0.4538	-0.1117	-0.1766	-0.0234	-0.0610	-0.0010	0.1016	0.0040	0.3852
soil_conse~n	0.7862	-0.0574	-0.0043	0.1094	-0.2515	0.0009	0.0955	0.0494	-0.0189	0.2914
tolerant_v~s	0.7097	0.3161	0.1197	-0.0355	0.0451	0.0359	0.0941	-0.1762	-0.0104	0.3374

.

```
. factor problem_identify farm_manage pest_disease weather_info new_practice input_provision general_advise
(obs=261)

Factor analysis/correlation                        Number of obs    =        261
    Method: principal factors                      Retained factors =          2
    Rotation: (unrotated)                          Number of params =         13
```

Factor	Eigenvalue	Difference	Proportion	Cumulative
Factor1	3.47407	3.30583	1.0813	1.0813
Factor2	0.16825	0.17206	0.0524	1.1337
Factor3	-0.00382	0.03396	-0.0012	1.1325
Factor4	-0.03778	0.02979	-0.0118	1.1207
Factor5	-0.06757	0.06819	-0.0210	1.0997
Factor6	-0.13576	0.04876	-0.0423	1.0574
Factor7	-0.18451	.	-0.0574	1.0000

```
    LR test: independent vs. saturated:  chi2(21) =  768.94 Prob>chi2 = 0.0000

Factor loadings (pattern matrix) and unique variances
```

Variable	Factor1	Factor2	Uniqueness
problem_id~y	0.7573	-0.0053	0.4264
farm_manage	0.7646	-0.1344	0.3973
pest_disease	0.7375	-0.1774	0.4246
weather_info	0.6759	0.2502	0.4805
new_practice	0.6677	-0.0101	0.5541
input_prov~n	0.6829	0.2106	0.4893
general_ad~e	0.6347	-0.1079	0.5855

```
Factor analysis/correlation                        Number of obs    =        261
    Method: principal factors                      Retained factors =          2
    Rotation: (unrotated)                          Number of params =          6
```

Factor	Eigenvalue	Difference	Proportion	Cumulative
Factor1	1.31431	1.20440	1.2881	1.2881
Factor2	0.10990	0.29232	0.1077	1.3959
Factor3	-0.18242	0.03907	-0.1788	1.2171
Factor4	-0.22149	.	-0.2171	1.0000

```
    LR test: independent vs. saturated:  chi2(6)  =  173.47 Prob>chi2 = 0.0000
```

Factor loadings (pattern matrix) and unique variances

Variable	Factor1	Factor2	Uniqueness
Radio	0.5143	0.1755	0.7047
Television	0.4999	0.1847	0.7160
Magazine	0.6683	-0.1145	0.5403
Newspaper	0.5944	-0.1785	0.6149

.

iii. Horn's Parallel Analysis

```
. paran Temprature Rainpattern GHgas Seasonchange ,iterations (1000)
(obs=255)
```

(principal components; 4 components retained)

Component	Eigenvalue	Difference	Proportion	Cumulative
1	2.24751	1.43703	0.5619	0.5619
2	0.81047	0.30588	0.2026	0.7645
3	0.50460	0.06718	0.1261	0.8906
4	0.43742	.	0.1094	1.0000

Variable	Eigenvectors 1	2	3	4
Temprature	0.54487	0.18345	0.43378	-0.69376
Rainpattern	0.52248	-0.26370	-0.79554	-0.15680
GHgas	0.47852	-0.60433	0.42022	0.47876
Seasonchange	0.44850	0.72910	-0.04857	0.51468

Computing: 10% 20% 30% 40% 50% 60% 70% 80% 90% 100%

Results of Horn's Parallel Analysis for principal components
1000 iterations, using the mean estimate

Component or Factor	Adjusted Eigenvalue	Unadjusted Eigenvalue	Estimated Bias
1	2.0915989	2.2475092	.15591037

Criterion: retain adjusted components > 1

```
. paran late_rain unevenspace_rain uneventemp_rain Insuff_rain lossharvest_rain cropfail_rain ,iterations (1000)
(obs=252)

            (principal components; 6 components retained)
Component     Eigenvalue      Difference     Proportion     Cumulative
------------------------------------------------------------------------------

     1          2.70093          1.78396        0.4502          0.4502
     2          0.91697          0.03696        0.1528          0.6030
     3          0.88002          0.25050        0.1467          0.7497
     4          0.62951          0.08932        0.1049          0.8546
     5          0.54019          0.20782        0.0900          0.9446
     6          0.33237                .        0.0554          1.0000

              Eigenvectors
    Variable |        1          2          3          4          5          6
-------------+------------------------------------------------------------------
             |
   late_rain |  0.43572   -0.12529    0.24771   -0.53807    0.65491    0.12105
unevenspac~n |  0.44807   -0.47975   -0.21276    0.29340   -0.18573    0.63499
uneventemp~n |  0.49118   -0.37868   -0.08252    0.14379   -0.10962   -0.75884
 Insuff_rain |  0.30570    0.31586    0.77269    0.44852   -0.07637    0.05225
lossharves~n |  0.32281    0.58289   -0.53054    0.35126    0.38856   -0.01381
cropfail_r~n |  0.41287    0.41377   -0.08965   -0.52836   -0.60644    0.05799

Computing: 10% 20% 30% 40% 50% 60% 70% 80% 90% 100%


Results of Horn's Parallel Analysis for principal components
1000 iterations, using the mean estimate

--------------------------------------------------
Component   Adjusted    Unadjusted    Estimated
or Factor   Eigenvalue  Eigenvalue    Bias
--------------------------------------------------
 1          2.4944042   2.7009345     .20653033
--------------------------------------------------
Criterion: retain adjusted components > 1
```

```
. paran waterharvesting onfarm_terrace contour_ploughing fallowing crop_rotation animal_manure improved_stove early_mature high_yielding replace_
> unproduct organic_pesticide store_seeds replanting Earling_warning adjusting_plant soil_conservation tolerant_varities ,iterations (1000)
(obs=259)
```

(principal components; 17 components retained)

Component	Eigenvalue	Difference	Proportion	Cumulative
1	7.86696	6.52393	0.4628	0.4628
2	1.34302	0.30694	0.0790	0.5418
3	1.03608	0.06386	0.0609	0.6027
4	0.97222	0.13187	0.0572	0.6599
5	0.84035	0.06163	0.0494	0.7093
6	0.77872	0.16013	0.0458	0.7551
7	0.61859	0.01248	0.0364	0.7915
8	0.60611	0.08337	0.0357	0.8272
9	0.52273	0.05828	0.0307	0.8579
10	0.46446	0.05363	0.0273	0.8852
11	0.41083	0.08589	0.0242	0.9094
12	0.32494	0.00960	0.0191	0.9285
13	0.31534	0.04371	0.0185	0.9471
14	0.27163	0.04098	0.0160	0.9631
15	0.23065	0.02523	0.0136	0.9766
16	0.20542	0.01348	0.0121	0.9887
17	0.19195	.	0.0113	1.0000

Eigenvectors

Variable	1	2	3	4	5	6
waterharve~g	0.25582	-0.13180	-0.04108	0.01446	-0.35216	-0.08555
onfarm_ter~e	0.26894	-0.36529	-0.01914	-0.08671	0.00337	0.15363
contour_pl~g	0.18691	-0.23645	0.42131	0.25685	0.40573	0.28843
fallowing	0.25526	-0.24124	-0.20854	-0.14655	0.21917	0.19822
crop_rotat~n	0.27600	0.08152	0.15089	-0.40020	-0.02527	-0.02758
animal_man~e	0.25509	-0.12161	0.27919	-0.37420	-0.15840	-0.03419
improved_s~e	0.17956	-0.34964	-0.41490	-0.03606	0.36872	0.01294
early_mature	0.28646	0.10562	-0.09842	0.21122	0.06444	0.06982
high_yield~g	0.29439	0.01704	-0.00464	0.18450	-0.02683	0.02176
replace_un~t	0.19695	-0.18411	0.08122	0.55780	-0.29423	-0.36473
organic_pe~e	0.18336	0.17644	-0.32033	0.02062	-0.46932	0.60887
store_seeds	0.26722	-0.02752	0.22017	-0.25563	-0.00163	-0.33435
replanting	0.16063	0.37033	0.47579	0.17608	0.12887	0.31482
Earling_wa~g	0.23251	0.25829	-0.26760	0.19800	0.25425	-0.29184
adjusting_~t	0.21822	0.47485	-0.13944	-0.24485	0.16582	-0.15608
soil_conse~n	0.28530	-0.04471	0.05201	0.02268	-0.25952	-0.12232
tolerant_v~s	0.25928	0.28779	-0.12872	0.13072	0.11762	-0.00213

Eigenvectors

Variable	7	8	9	10	11	12
waterharve~g	0.60640	-0.00078	0.04509	0.03820	-0.26772	0.06648
onfarm_ter~e	0.13293	-0.08603	-0.29588	0.10161	0.18717	0.41897
contour_pl~g	0.04745	-0.34545	0.36900	-0.22042	-0.09278	0.10991
fallowing	-0.02402	-0.08131	-0.48709	0.11536	0.31004	-0.34582
crop_rotat~n	-0.15828	-0.01587	0.17983	0.19129	-0.05092	-0.29032
animal_man~e	-0.26862	0.02863	0.11831	0.35684	-0.04818	0.40596
improved_s~e	0.06649	0.54509	0.37298	0.04586	-0.21415	-0.09596
early_mature	-0.21809	0.03560	-0.32423	-0.17543	-0.38266	0.10658
high_yield~g	-0.33843	0.04073	-0.15123	-0.35634	-0.06659	0.03504
replace_un~t	-0.26660	0.24584	0.02357	0.22083	0.13168	-0.00522
organic_pe~e	-0.13468	0.01470	0.34459	-0.13643	0.23891	0.01176
store_seeds	-0.10132	0.07599	0.14367	-0.42541	0.27466	-0.26790
replanting	0.27946	0.51155	-0.13764	0.19670	0.16915	-0.11273
Earling_wa~g	0.17992	-0.21485	0.21526	0.11362	0.52675	0.22218
adjusting_~t	0.07523	0.14134	-0.09685	-0.22098	-0.15231	0.36090
soil_conse~n	0.34639	-0.18528	-0.06174	-0.19912	-0.09784	-0.28229
tolerant_v~s	-0.11159	-0.37007	0.07179	0.46272	-0.31339	-0.27118

Eigenvectors

Variable	13	14	15	16	17
waterharve~g	-0.25635	0.41905	0.27056	0.14754	-0.01871
onfarm_ter~e	0.03632	-0.17374	-0.01107	-0.49664	-0.38852
contour_pl~g	0.16518	0.19978	-0.01832	-0.00541	0.15262
fallowing	0.16850	0.28818	0.05231	0.28081	0.24054
crop_rotat~n	-0.33746	0.26183	-0.36308	-0.46028	0.16007
animal_man~e	0.03487	-0.21015	0.04217	0.47118	0.15907
improved_s~e	0.02727	-0.16718	-0.06351	0.04135	-0.06055
early_mature	-0.28195	-0.26104	0.30196	-0.19992	0.47132
high_yield~g	-0.34223	0.15501	-0.33671	0.34877	-0.47949
replace_un~t	0.30564	0.22746	-0.06113	-0.16481	0.12542
organic_pe~e	0.12260	-0.00224	0.08978	-0.04490	0.05653
store_seeds	0.06407	-0.11153	0.53506	-0.07198	-0.17129
replanting	-0.05810	-0.11365	0.01676	0.04121	-0.08335
Earling_wa~g	-0.32536	-0.11301	-0.06557	0.08660	0.15346
adjusting_~t	0.49554	0.29950	-0.13332	-0.06549	0.03195
soil_conse~n	0.24984	-0.50067	-0.45354	0.08731	0.13278
tolerant_v~s	0.18727	-0.09042	0.23508	0.00666	-0.40433

Computing: 10% 20% 30% 40% 50% 60% 70% 80% 90% 100%

Results of Horn's Parallel Analysis for principal components
1000 iterations, using the mean estimate

Component or Factor	Adjusted Eigenvalue	Unadjusted Eigenvalue	Estimated Bias
1	7.4083692	7.8669564	.45858717
2	.93623155	1.3430236	.40679204
3	.70747113	1.0360823	.32861114

Criterion: retain adjusted components > 1

.

```
. paran problem_identify farm_manage pest_disease weather_info new_practice input_provision general_advise ,iterations (1000)
(obs=261)

            (principal components; 7 components retained)
Component    Eigenvalue     Difference    Proportion    Cumulative
------------------------------------------------------------------

     1        4.00869         3.29349      0.5727         0.5727
     2        0.71520         0.13636      0.1022         0.6748
     3        0.57884         0.04497      0.0827         0.7575
     4        0.53387         0.08421      0.0763         0.8338
     5        0.44966         0.08040      0.0642         0.8980
     6        0.36926         0.02478      0.0528         0.9508
     7        0.34448               .      0.0492         1.0000

               Eigenvectors
    Variable |      1          2          3          4          5          6
-------------+------------------------------------------------------------------

problem_id~y |   0.40092   -0.01430   -0.20593   -0.11922   -0.65818   -0.57821
 farm_manage |   0.40193   -0.22697   -0.22907   -0.39913    0.11349    0.16450
pest_disease |   0.39113   -0.35014   -0.20018   -0.26006    0.50898   -0.04874
weather_info |   0.36437    0.63102   -0.05080   -0.21389   -0.19587    0.57186
new_practice |   0.36529   -0.05126   -0.38753    0.82720    0.05286    0.14659
input_prov~n |   0.36907    0.47971    0.43185    0.10806    0.43449   -0.46404
general_ad~e |   0.34975   -0.44130    0.72512    0.13101   -0.25478    0.26907

               Eigenvectors
    Variable |      7
-------------+-----------

problem_id~y |   0.12210
 farm_manage |  -0.73159
pest_disease |   0.59605
weather_info |   0.23521
new_practice |  -0.07220
input_prov~n |  -0.17718
general_ad~e |   0.05152

Computing: 10% 20% 30% 40% 50% 60% 70% 80% 90% 100%


Results of Horn's Parallel Analysis for principal components
1000 iterations, using the mean estimate

--------------------------------------------------
Component   Adjusted    Unadjusted    Estimated
or Factor   Eigenvalue  Eigenvalue    Bias
--------------------------------------------------
 1          3.802926    4.0086945     .20576847
--------------------------------------------------
Criterion: retain adjusted components > 1
```

```
. paran Radio Television Magazine Newspaper ,iterations (1000)
(obs=261)

            (principal components; 4 components retained)
Component     Eigenvalue     Difference    Proportion    Cumulative
-------------------------------------------------------------------

     1         2.04765         1.17989       0.5119         0.5119
     2         0.86776         0.24700       0.2169         0.7289
     3         0.62076         0.15693       0.1552         0.8840
     4         0.46383               .       0.1160         1.0000

               Eigenvectors
    Variable |       1          2          3          4
-------------+-----------------------------------------
       Radio |   0.47370    0.49339   -0.72484    0.08233
  Television |   0.46356    0.54849    0.68809    0.10389
    Magazine |   0.55234   -0.36169    0.02952   -0.75049
   Newspaper |   0.50561   -0.57000    0.01597    0.64746

Computing: 10% 20% 30% 40% 50% 60% 70% 80% 90% 100%


Results of Horn's Parallel Analysis for principal components
1000 iterations, using the mean estimate

--------------------------------------------------
Component   Adjusted    Unadjusted    Estimated
or Factor   Eigenvalue  Eigenvalue    Bias
--------------------------------------------------
 1          1.9330371   2.0476469     .11460984
--------------------------------------------------
Criterion: retain adjusted components > 1
```

Supplementary Material (S3): Full Mediation Regression Results

Results

GLM Mediation Model

```
jamm::jammGLM(
  formula = list( Coping ~ Shade + Extension + Media + Education + Agexp + Heat + Altitude + Offarm,
        Threat ~ Shade + Extension + Media + Education + Agexp + Heat + Altitude + Offarm,
        Perception ~ Coping + Threat + Shade + Extension + Media + Education + Agexp + Heat + Altitude + Offarm ),
  data = data,
  ciType = "bca",
  bootN = 5000,
  tableOptions = c("beta", "component", "regression"))
```

Models Info

**Mediators Models**		
	**m1**	**Coping ~ Shade + Extension + Media + Education + Agexp + Heat + Altitude + Offarm**
	**m2**	**Threat ~ Shade + Extension + Media + Education + Agexp + Heat + Altitude + Offarm**
**Full Model**		
	**m3**	**Perception ~ Coping + Threat + Shade + Extension + Media + Education + Agexp + Heat + Altitude + Offarm**
**Indirect Effects**		
	**IE 1**	**Shade ⇒ Coping ⇒ Perception**
	**IE 2**	**Shade ⇒ Threat ⇒ Perception**
	**IE 3**	**Extension ⇒ Coping ⇒ Perception**

Models Info

	IE 4	Extension ⇒ Threat ⇒ Perception
	IE 5	Media ⇒ Coping ⇒ Perception
	IE 6	Media ⇒ Threat ⇒ Perception
	IE 7	Education ⇒ Coping ⇒ Perception
	IE 8	Education ⇒ Threat ⇒ Perception
	IE 9	Agexp ⇒ Coping ⇒ Perception
	IE 10	Agexp ⇒ Threat ⇒ Perception
	IE 11	Heat ⇒ Coping ⇒ Perception
	IE 12	Heat ⇒ Threat ⇒ Perception
	IE 13	Altitude ⇒ Coping ⇒ Perception
	IE 14	Altitude ⇒ Threat ⇒ Perception
	IE 15	Offarm ⇒ Coping ⇒ Perception
	IE 16	Offarm ⇒ Threat ⇒ Perception
Sample size	N	245

Path Model

Conceptual Diagram

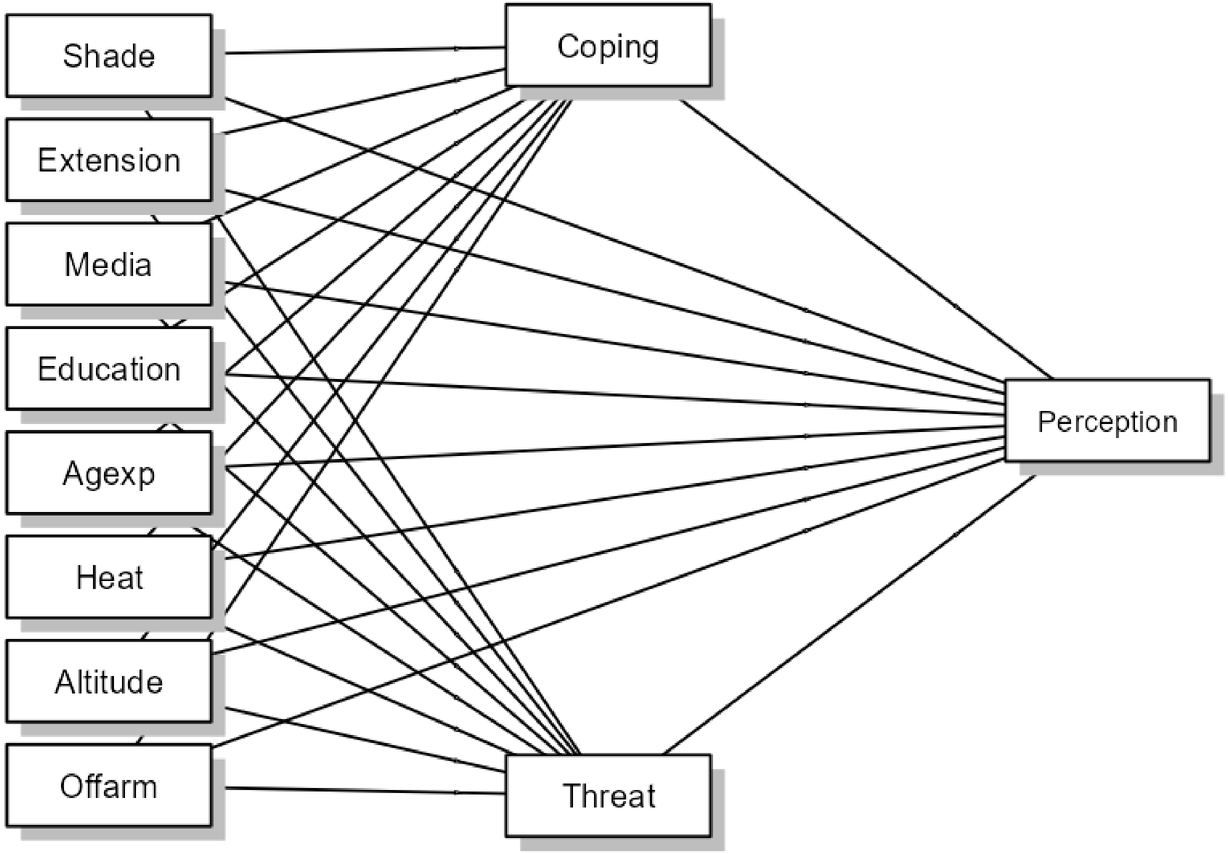
Shade
Extension
Media
Education
Agexp
Heat
Altitude
Offarm
Coping
Threat
Perception

Diagram notes
Covariances among IV are estimated but not shown

Mediation

Indirect and Total Effects

				95% C.I. (a)				
**Type**	**Effect**	Estimate	SE	Lower	Upper	β	z	p
**Indirect**	**Shade ⇒ Coping ⇒ Perception**	-1.20e−4	0.00108	-0.00619	0.00323	-3.04e−4	-0.111	0.911
	**Shade ⇒ Threat ⇒ Perception**	-0.00544	0.00479	-0.01794	0.00214	-0.01378	-1.134	0.257
	**Extension ⇒ Coping ⇒ Perception**	0.01619	0.02162	-0.02961	0.07051	0.02061	0.749	0.454
	**Extension ⇒ Threat ⇒ Perception**	0.01217	0.01020	-0.00362	0.03919	0.01549	1.193	0.233
	**Media ⇒ Coping ⇒ Perception**	0.00447	0.00640	-0.00626	0.03352	0.00549	0.699	0.485
	**Media ⇒ Threat ⇒ Perception**	0.03714	0.01544	0.01191	0.07925	0.04564	2.406	0.016
	**Education ⇒ Coping ⇒ Perception**	1.26e-4	1.81e-4	-1.84e−4	8.34e-4	0.00516	0.698	0.485
	**Education ⇒ Threat ⇒ Perception**	**5.04e-4**	**3.27e-4**	4.98e-5	**0.00127**	**0.02065**	**1.542**	**0.123**
	**Agexp ⇒ Coping ⇒ Perception**	**-7.27e−6**	**6.70e-5**	**-3.63e−4**	**1.71e-4**	**-2.97e−4**	**-0.108**	**0.914**
	**Agexp ⇒ Threat ⇒ Perception**	**8.22e-4**	**3.81e-4**	**2.25e-4**	**0.00179**	**0.03355**	**2.159**	**0.031**
	**Heat ⇒ Coping ⇒ Perception**	**-5.63e−4**	**0.00132**	**-0.01046**	**0.00206**	**-0.00142**	**-0.427**	**0.669**
	**Heat ⇒ Threat ⇒ Perception**	**0.00913**	**0.00539**	**-3.67e−5**	**0.02539**	**0.02307**	**1.693**	**0.090**

Indirect and Total Effects

Type	Effect	Estimate	SE	95% C.I. (a) Lower	Upper	β	z	p
	Altitude ⇒ Coping ⇒ Perception	-7.75e−4	0.00268	-0.01572	0.00482	-8.87e−4	-0.289	0.773
	Altitude ⇒ Threat ⇒ Perception	-0.00782	0.01068	-0.03757	0.01485	-0.00895	-0.732	0.464
	Offarm ⇒ Coping ⇒ Perception	0.00152	0.00271	-0.00300	0.01864	0.00224	0.559	0.576
	Offarm ⇒ Threat ⇒ Perception	0.00118	0.00762	-0.01068	0.01544	0.00174	0.154	0.877
Component	Shade ⇒ Coping	-0.00360	0.03194	-0.07085	0.05940	-0.00645	-0.113	0.910
	Coping ⇒ Perception	0.03337	0.04432	-0.06395	0.13841	0.04720	0.753	0.451
	Shade ⇒ Threat	-0.02532	0.02083	-0.06624	0.01499	-0.07505	-1.215	0.224
	Threat ⇒ Perception	0.21476	0.06795	0.07186	0.36220	0.18360	3.161	0.002
	Extension ⇒ Coping	0.48510	0.06745	0.34938	0.60582	0.43666	7.192	<.001
	Extension ⇒ Threat	0.05667	0.04400	-0.02918	0.14395	0.08438	1.288	0.198
	Media ⇒ Coping	0.13389	0.07144	-0.03691	0.29207	0.11634	1.874	0.061
	Media ⇒ Threat	0.17296	0.04660	0.07159	0.27078	0.24861	3.711	<.001
	Education ⇒ Coping	0.00378	0.00204	-2.08e−4	0.00795	0.10938	1.854	0.064
	Education ⇒ Threat	0.00235	0.00133	-1.89e−4	0.00478	0.11249	1.767	0.077
	Agexp ⇒ Coping	-2.18e−4	0.00199	-0.00414	0.00316	-0.00628	-0.110	0.913
	Agexp ⇒ Threat	0.00383	0.00130	0.00150	0.00641	0.18275	2.955	0.003

Indirect and Total Effects

Type	Effect	Estimate	SE	95% C.I. (a) Lower	Upper	β	z	p
	Heat ⇒ Coping	-0.01687	0.03250	-0.09038	0.05422	-0.03015	-0.519	0.604
	Heat ⇒ Threat	0.04251	0.02120	-0.00397	0.08515	0.12565	2.005	0.045
	Altitude ⇒ Coping	-0.02321	0.07417	-0.14671	0.13980	-0.01878	-0.313	0.754
	Altitude ⇒ Threat	-0.03642	0.04839	-0.14515	0.07354	-0.04875	-0.753	0.452
	Offarm ⇒ Coping	0.04542	0.05433	-0.08389	0.15403	0.04748	0.836	0.403
	Offarm ⇒ Threat	0.00548	0.03544	-0.04853	0.06173	0.00948	0.155	0.877
Direct	Shade ⇒ Perception	-0.04401	0.02222	-0.09154	3.34e-4	-0.11153	-1.981	0.048
	Extension ⇒ Perception	0.25438	0.05161	0.12506	0.37309	0.32384	4.929	<.001
	Media ⇒ Perception	0.12646	0.05125	0.00617	0.25737	0.15540	2.467	0.014
	Education ⇒ Perception	0.00299	0.00143	5.29e-5	0.00578	0.12226	2.085	0.037
	Agexp ⇒ Perception	0.00111	0.00140	-0.00136	0.00371	0.04529	0.792	0.429
	Heat ⇒ Perception	0.02025	0.02274	-0.02615	0.06514	0.05118	0.891	0.373
	Altitude ⇒ Perception	-0.15695	0.05152	-0.26750	-0.04515	-0.17965	-3.046	0.002
	Offarm ⇒ Perception	-0.07241	0.03774	-0.14073	0.00128	-0.10706	-1.918	0.055
Total	Shade ⇒ Perception	-0.04891	0.02207	-0.09640	-0.00258	-0.12508	-2.216	0.027
	Extension ⇒ Perception	0.27817	0.04708	0.16692	0.38673	0.35459	5.909	<.001

Indirect and Total Effects

				95% C.I. (a)				
**Type**	**Effect**	Estimate	SE	Lower	Upper	β	z	p
	**Media ⇒ Perception**	**0.16878**	**0.04932**	**0.05204**	**0.29739**	**0.20953**	**3.422**	**<.001**
	**Education ⇒ Perception**	**0.00346**	**0.00141**	**6.07e-4**	**0.00626**	**0.14213**	**2.453**	**0.014**
	**Agexp ⇒ Perception**	**0.00202**	**0.00139**	**-5.59e−4**	**0.00445**	**0.08201**	**1.453**	**0.146**
	**Heat ⇒ Perception**	**0.03187**	**0.02245**	**-0.01626**	**0.07641**	**0.08119**	**1.419**	**0.156**
	**Altitude ⇒ Perception**	**-0.16466**	**0.05196**	**-0.28146**	**-0.05520**	**-0.18719**	**-3.169**	**0.002**
	**Offarm ⇒ Perception**	**-0.06818**	**0.03816**	**-0.13494**	**0.01025**	**-0.10008**	**-1.787**	**0.074**

Note. Confidence intervals computed with method: Bias corrected bootstrap

Note. Betas are completely standardized effect sizes

Regressions Results

Total effects

ANOVA Table

R-squared	F	df1	df2	p
0.275	11.6	8.00	245	<.001

Total effects predicting: Perception

Names	Effect	Estimate	SE	Lower	Upper	β	df	t	p
Shade	Shade	-0.04891	0.02243	-0.0931	-0.00474	-0.1253	245	-2.18	0.030
Extension	Extension	0.27817	0.04784	0.1839	0.37241	0.3580	245	5.81	<.001
Media	Media	0.16878	0.05012	0.0701	0.26751	0.2097	245	3.37	<.001
Education	Education	0.00346	0.00143	6.38e-4	0.00629	0.1415	245	2.41	0.016
Agexp	Agexp	0.00202	0.00141	-7.61e−4	0.00480	0.0818	245	1.43	0.154
Heat	Heat	0.03187	0.02281	-0.0131	0.07680	0.0814	245	1.40	0.164
Altitude	Altitude	-0.16466	0.05280	-0.2687	-0.06065	-0.1886	245	-3.12	0.002
Offarm	Offarm	-0.06818	0.03878	-0.1446	0.00820	-0.1010	245	-1.76	0.080

Mediators Models

Dependent variable: Coping

ANOVA

R-squared	F	df1	df2	p
0.274	11.7	8.00	249	<.001

Regression

Names	Effect	Estimate	SE	Lower	Upper	β	df	t	p
Shade	Shade	-0.01665	0.03162	-0.07893	0.04563	-0.02988	249	-0.526	0.599
Extension	Extension	0.48900	0.06738	0.35629	0.62170	0.44097	249	7.258	<.001
Media	Media	0.11815	0.07136	-0.02240	0.25870	0.10286	249	1.656	0.099
Education	Education	0.00358	0.00204	-4.43e−4	0.00761	0.10261	249	1.753	0.081
Agexp	Agexp	-2.91e−4	0.00200	-0.00424	0.00366	-0.00827	249	-0.145	0.885
Heat	Heat	-0.00490	0.03236	-0.06864	0.05885	-0.00877	249	-0.151	0.880
Altitude	Altitude	-0.03422	0.07451	-0.18096	0.11253	-0.02746	249	-0.459	0.646
Offarm	Offarm	0.05354	0.05518	-0.05514	0.16222	0.05555	249	0.970	0.333

Dependent variable: Threat

ANOVA

R-squared	F	df1	df2	p
0.146	5.16	8.00	242	<.001

Regression

Names	Effect	Estimate	SE	Lower	Upper	β	df	t	p
Shade	Shade	-0.02440	0.02097	-0.06571	0.01691	-0.0729	242	-1.164	0.246
Extension	Extension	0.04528	0.04433	-0.04204	0.13260	0.0679	242	1.021	0.308
Media	Media	0.16265	0.04734	0.06941	0.25590	0.2356	242	3.436	<.001
Education	Education	0.00204	0.00135	-6.13e−4	0.00469	0.0971	242	1.514	0.131
Agexp	Agexp	0.00352	0.00131	9.30e-4	0.00611	0.1663	242	2.678	0.008
Heat	Heat	0.04921	0.02137	0.00712	0.09131	0.1466	242	2.303	0.022
Altitude	Altitude	-0.03959	0.04886	-0.13583	0.05666	-0.0529	242	-0.810	0.419
Offarm	Offarm	0.00675	0.03616	-0.06449	0.07798	0.0116	242	0.187	0.852

Full model effects

ANOVA Table

R-squared	F	df1	df2	p
0.306	10.3	10.0	234	<.001

Full model predicting Perception

Names	Effect	Estimate	SE	Lower	Upper	β	df	t	p
Coping	Coping	0.03337	0.04535	-0.05598	0.12273	0.0476	234	0.736	0.463
Threat	Threat	0.21476	0.06952	0.07778	0.35173	0.1842	234	3.089	0.002
Shade	Shade	-0.04401	0.02274	-0.08881	7.83e-4	-0.1127	234	-1.936	0.054
Extension	Extension	0.25438	0.05281	0.15033	0.35843	0.3274	234	4.817	<.001
Media	Media	0.12646	0.05244	0.02313	0.22978	0.1571	234	2.411	0.017
Education	Education	0.00299	0.00147	9.88e-5	0.00587	0.1221	234	2.038	0.043
Agexp	Agexp	0.00111	0.00143	-0.00172	0.00394	0.0450	234	0.774	0.440
Heat	Heat	0.02025	0.02327	-0.02559	0.06609	0.0518	234	0.870	0.385
Altitude	Altitude	-0.15695	0.05272	-0.26082	-0.05309	-0.1798	234	-2.977	0.003
Offarm	Offarm	-0.07241	0.03862	-0.14850	0.00368	-0.1072	234	-1.875	0.062

Supplementary Material (S4): Multicollinearity, Heteroskedasticity and Normality Tests

```
. reg Perception Coping Threat Shade Extension Media Education Agexp Heat Altitude Offarm

      Source |       SS           df       MS      Number of obs   =       245
-------------+----------------------------------   F(10, 234)      =     10.32
       Model |  2.91797442        10  .291797442   Prob > F        =    0.0000
    Residual |  6.61645023       234  .028275428   R-squared       =    0.3060
-------------+----------------------------------   Adj R-squared   =    0.2764
       Total |  9.53442465       244  .039075511   Root MSE        =    .16815

------------------------------------------------------------------------------
  Perception |      Coef.   Std. Err.      t    P>|t|     [95% Conf. Interval]
-------------+----------------------------------------------------------------
      Coping |   .0333727   .0453543     0.74   0.463    -.0559822    .1227276
      Threat |   .2147578   .0695241     3.09   0.002     .0777847    .3517309
       Shade |  -.0440118   .0227366    -1.94   0.054    -.0888065    .0007829
   Extension |   .2543827   .0528131     4.82   0.000     .1503327    .3584326
       Media |   .1264583   .0524446     2.41   0.017     .0231344    .2297823
   Education |   .0029865   .0014657     2.04   0.043     .0000988    .0058741
       Agexp |   .0011099   .0014348     0.77   0.440    -.0017169    .0039367
        Heat |   .0202521   .0232681     0.87   0.385    -.0255897    .0660939
    Altitude |  -.1569527   .0527182    -2.98   0.003    -.2608157   -.0530896
      Offarm |  -.0724098   .0386204    -1.87   0.062    -.1484979    .0036784
       _cons |   1.409679   .4069027     3.46   0.001     .6080184     2.21134
------------------------------------------------------------------------------

. hettest

Breusch-Pagan / Cook-Weisberg test for heteroskedasticity 
         Ho: Constant variance
         Variables: fitted values of Perception

         chi2(1)      =     1.79
         Prob > chi2  =   0.1809

. vif

    Variable |       VIF       1/VIF  
-------------+----------------------
   Extension |      1.52    0.656045
       Media |      1.40    0.714028
      Coping |      1.39    0.720780
    Altitude |      1.23    0.814512
   Education |      1.21    0.823755
      Threat |      1.19    0.839456
        Heat |      1.17    0.857827
       Agexp |      1.16    0.865221
       Shade |      1.12    0.893370
      Offarm |      1.10    0.909621
-------------+----------------------
    Mean VIF |      1.25

.
```

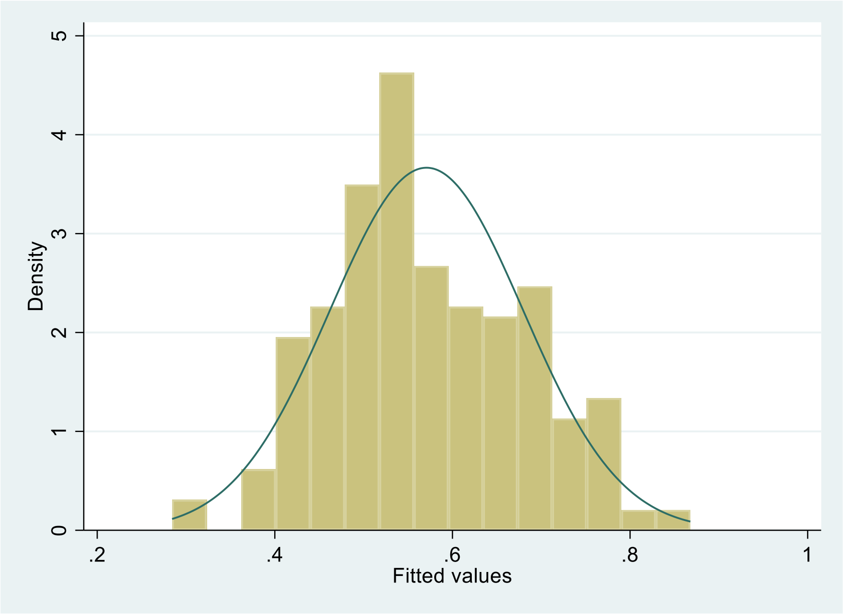


```
. swilk resid

                   Shapiro-Wilk W test for normal data

    Variable │        Obs       W           V         z       Prob>z
─────────────┼──────────────────────────────────────────────────────
       resid │        250    0.98658      2.434     2.070    0.01925
```

Supplementary Material (S5): Sensitivity Analysis Plots for Indirect Effects

Exposure: Media

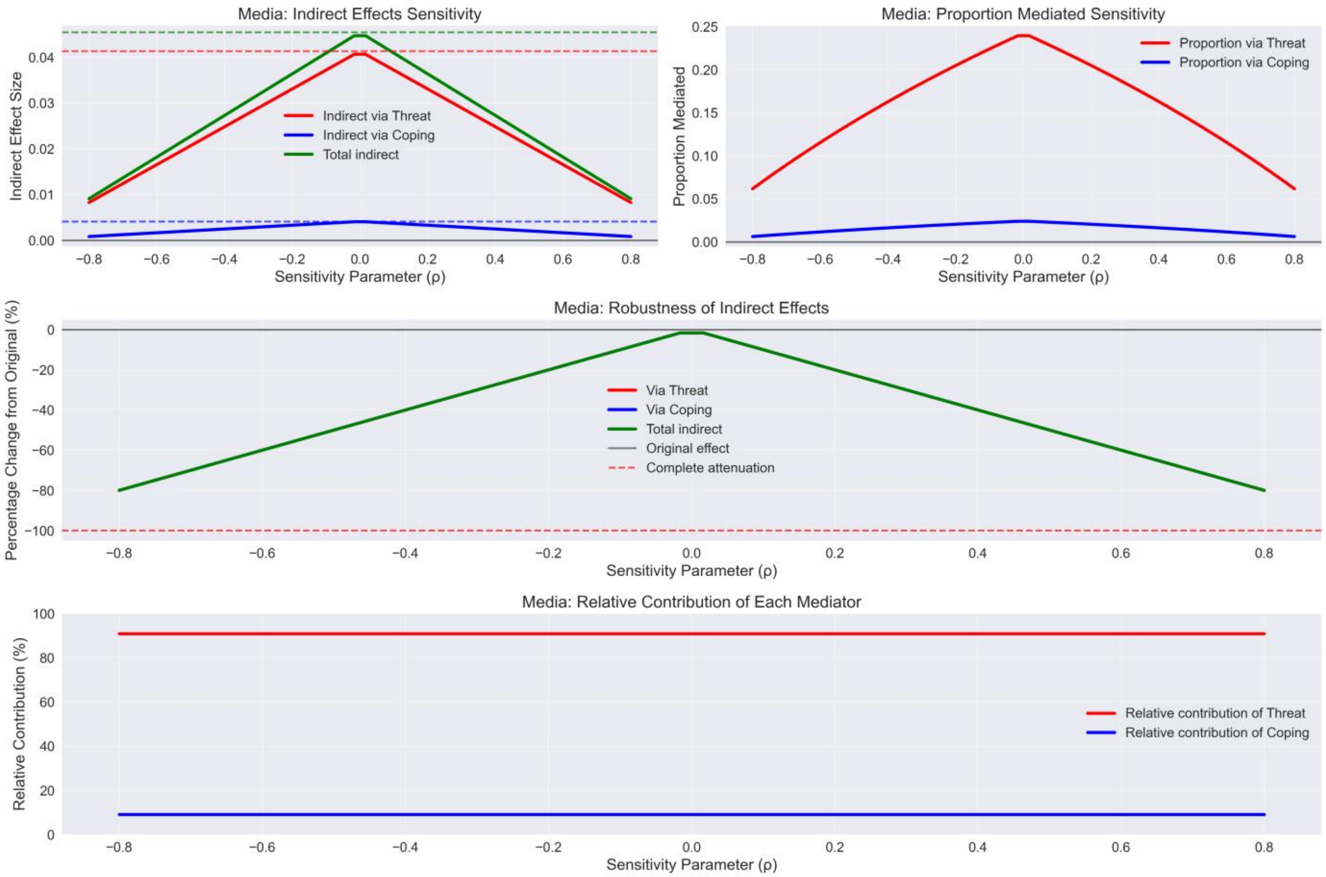

Figure: Sensitivity of indirect effects for Media to unmeasured confounding. Shows how effects through Threat and Coping change with different levels of confounding.

Exposure: Agexp

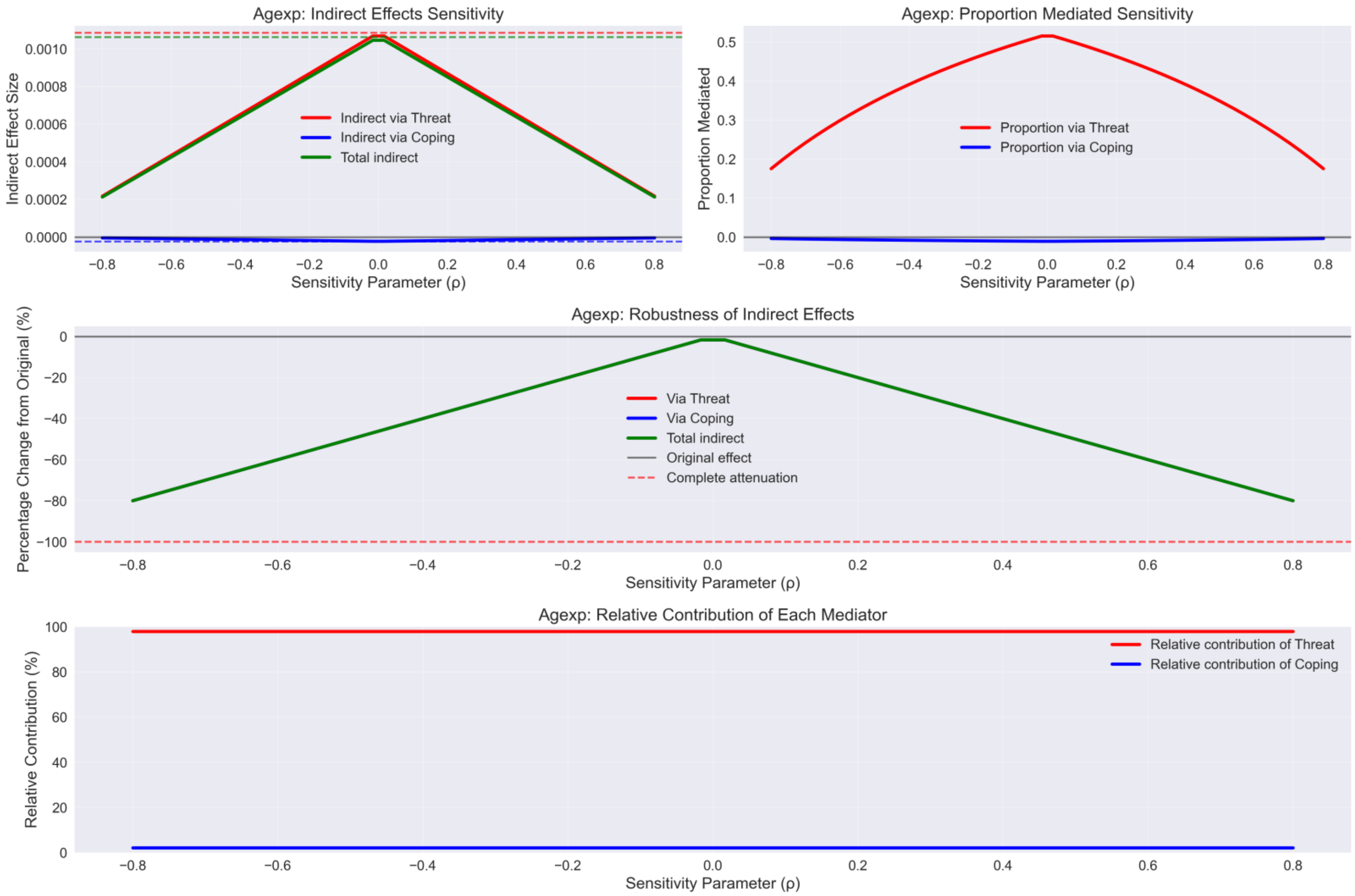

Figure: Sensitivity of indirect effects for Agexp to unmeasured confounding. Shows how effects through Threat and Coping change with different levels of confounding.

Exposure: Heat

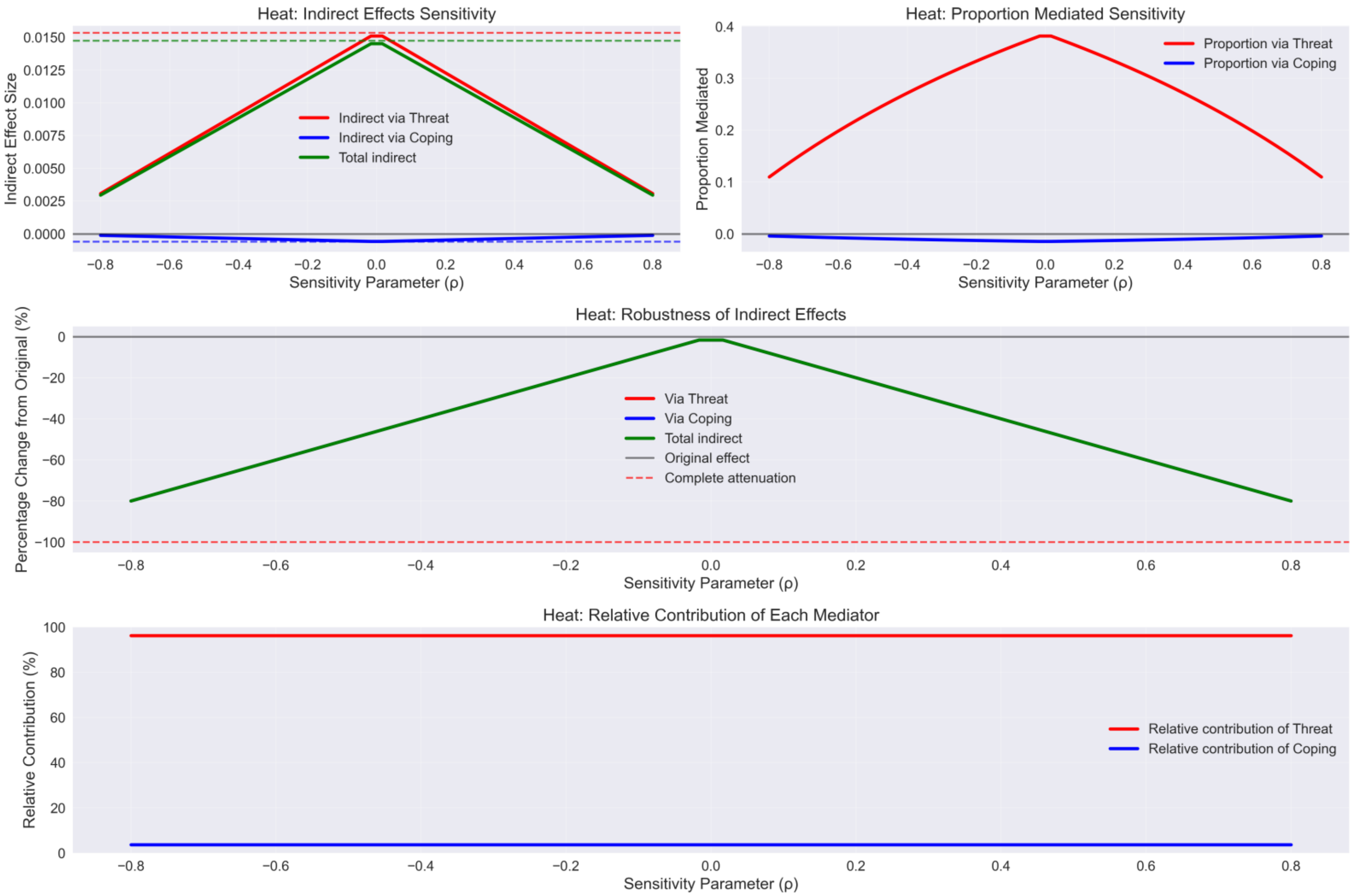

Figure: Sensitivity of indirect effects for Heat to unmeasured confounding. Shows how effects through Threat and Coping change with different levels of confounding
