# Supplementary material for: Factors Affecting Smallholders’ Perception of Climate Change in Eritrea
Source: Environ Manage. 2026 Jan 30;76(3):90. doi: 10.1007/s00267-026-02383-7 (PMC12858499; doi:10.1007/s00267-026-02383-7)
Supplement: Supplementary file 2 — Supplementary Materials 1_5 [file 267_2026_2383_MOESM2_ESM.pdf]

README

1. Purpose and Scope

- This dataset was prepared exclusively for editorial and peer-review purposes. It accompanies the manuscript submitted to Environmental Management titled: "Factors Affecting Smallholders' Perception of Climate Change in Eritrea."
- The file contains synthetically generated, anonymized variables that mirror the structure of the original dataset used in the analysis.
- No individual-level respondent data are included.

2. Method for Preparing the Dataset (Gaussian Copula Synthetic Data Generation)

- To ensure confidentiality, all data in this file were generated using a Gaussian Copula–based synthetic data procedure.
- This method preserves general statistical properties of the original dataset without reproducing any real record.

3. Variable Descriptions

Variable	Description	Coding / Derivation	Type
Perception	Farmers' perception of climate-related threats to production	Normalized factor score (F1) from factor analysis	Continuous (index, 0 -1)
Threat	Perceived climate-related and environmental threats	Normalized factor score (F1) from factor analysis	Continuous (index, 0 -1)
Coping	Farmers' coping capacity in response to climate impacts	Normalized factor score (F1) from factor analysis	Continuous (index, 0 -1)
Extension	Satisfaction with agricultural extension services	Index derived from Likert-scale items; higher = greater satisfaction	Continuous (index, 0 -1)
Media	Exposure to agricultural information via mass media	Index of exposure through radio/TV/magazines/newspapers	Continuous (index, 0 -1)
Education	Highest level of formal education (squared)	Ordinal scale 1–6 (1 = none, 6 = university/college)	Ordinal
Agexp	Combined age and farming experience	Product of age × experience	Continuous
Altitude	Elevation of farm location	Natural log of altitude (meters above sea level)	Continuous
Offarm	Household has off-farm income	1 = Yes, 0 = No	Binary
Heat	Observed heat stress among dairy cows	1 = Yes, 0 = No	Binary
Shade	Ownership of improved dairy shade structure	1 = Yes, 0 = No	Binary

4. Notes on Factor Scores and Normalization

- Perception, Threat, Coping, Extension, and Media were derived using factor analysis (performed in Stata 16)
- The factor analysis codes and the results with all items included is provided in Supplementary materials (S2)
- Scores were normalized to the 0–1 range using Min–Max scaling to improve comparability across constructs.

5. Analytical Replication Notes

- The mediation analysis was conducted using Jamovi version 2.6.44, employing Generalized Linear Model (GLM) mediation within the medmod module.
- All regression models were checked for multicollinearity, heteroskedasticity, and residual normality before estimation.
- The accompanying code reproduces all regression tables in the manuscript.

6. Data File Information

- Number of observations: 245
- Number of variables: 11
